# Statin Use and the Risk of Prostate Cancer Biochemical Recurrence Following Definitive Therapy: A Systematic Review and Meta-Analysis of Cohort Studies

**DOI:** 10.3389/fonc.2022.887854

**Published:** 2022-05-09

**Authors:** Jian-Xuan Sun, Chen-Qian Liu, Xing-Yu Zhong, Jin-Zhou Xu, Ye An, Meng-Yao Xu, Jia Hu, Zong-Biao Zhang, Qi-Dong Xia, Shao-Gang Wang

**Affiliations:** Department and Institute of Urology, Tongji Hospital, Tongji Medical College, Huazhong University of Science and Technology, Wuhan, China

**Keywords:** statins, prostate cancer, biochemical recurrence, meta‐analysis, radical prostatectomy, radiotherapy

## Abstract

**Background:**

Numerous studies have reported the role of statins on biochemical recurrence (BCR) among patients with prostate cancer (PCa) after definite treatment. However, the conclusions of these studies are contradictory. We aimed to determine the effect of statins on BCR of PCa using a systematic review and meta-analysis.

**Methods:**

We searched PubMed (Medline) and other databases for cohort studies evaluating the effect of statins on the BCR of patients with PCa between January 1, 2000, and December 31, 2021. The random effects (RE) model and quality effects (QE) model were used to calculate the pooled hazard ratio (pHR) and pooled risk ratio (pRR) and their 95% confidence interval (95% CI).

**Results:**

A total of 33 cohort studies were finally selected and included in this systematic review and meta-analysis. Statin use was significantly associated with a 14% reduction in the HR of BCR (pHR: 0.86, 95% CI: 0.78 to 0.95, I^2^ = 64%, random effects model, 31 studies) and a 26% reduction in the RR of BCR (pRR: 0.74, 95% CI: 0.57 to 0.94, 24,591 patients, I^2^ = 88%, random effects model, 15 studies) among patients with PCa. The subgroup analyses showed that statins could result in 22% reduction in the HR of BCR (pHR: 0.78, 95% CI: 0.61 to 0.98, I^2^ = 57%, random effects model) among patients accepting radiotherapy (RT).

**Conclusions:**

Our study suggests that statins have a unique role in the reduction of BCR in patients with PCa after definite treatment, especially RT. In the future, more clinical trials and *in vitro* and animal experiments are needed to further verify the effects of statins in PCa and the potential mechanisms.

## Introduction

Prostate cancer (PCa) has the second highest incidence and the fifth highest mortality among all the malignant tumors in men around the world, causing more than 1,600,000 new cases and approximately 366,000 deaths annually (1). According to the data provided by the Global Burden of Disease Database, in 2017, there were 144,887 newly diagnosed PCa and 51,718 deaths in China, and the incidence of PCa is increasing year by year, which brought a heavy burden to public health and the national economy (2). Despite the high incidence, patients with non-metastatic PCa could choose various treatments such as active surveillance, radical prostatectomy (RP) and pelvic lymph node dissection (PLND), radiotherapy (RT), and androgen deprivation therapy (ADT) according to the stage of disease and the prognosis is good for those with low risk PCa (3). After treatment with curative intent, the measurement of prostate-specific antigen (PSA) becomes the most validated and sensitive method to monitor relapse (4). Biochemical recurrence (BCR) is defined as the return of detectable PSA, and nearly 20%–40% men treated with RP (5) or 30%–50% of those treated with RT will develop BCR (6), which indicates a nearly 30% probability of clinical recurrence after RP (7) and approximately 16.4% probability of death (8). Since BCR is one of the strongest evidences for clinical recurrence and progression of PCa, it is urgent for us to find effective treatment and protective factors to decrease the risk of BCR and improve the survival of patients after primary treatments.

Statins are 3-hydroxy-3-methyl-glutaryl coenzyme A (HMG-CoA) reductase inhibitors, which could inhibit the cholesterol synthesis by suppressing the activity of the rate-limiting enzyme in the liver. As commonly used drugs for secondary prevention of cardiovascular disease, statins are widely used worldwide. A cross-sectional study based on a total of 2,613,035 participants in 31 provinces in China showed that about 19.3% of them had ever used or were using statins (9). Although the role of statins in preventing cardiovascular disease by improving hypercholesterolemia is indisputable, in recent years, increasing evidence has suggested that statins also play a non-negligible role in chemoprevention and treatment of other diseases such as erectile dysfunction possibly by improving hyperhomocysteinemia (10, 11), and advanced tumors, including colon cancer, pancreatic cancer, and PCa (12–17). Existing studies have shown that the effects of statins on PCa are mainly achieved through two kinds of mechanisms: cholesterol-mediated and non-cholesterol-mediated pathways (18). Statins could influence the growth and progression of PCa mainly by cholesterol-mediated pathways. A positive correlation between cholesterol accumulation in prostate tissue and PCa incidence was reported as early as 1981 (19). Several mechanisms have demonstrated that dysregulation of cholesterol homeostasis in prostate cells contributes to the development of PCa. One study found that hypermethylation of the ABCA1 promoter resulted in a decreased expression of cholesterol efflux transporters, resulting in lower cholesterol efflux rates and increased cholesterol levels in prostate cancer cells. The presence of this epigenetic alteration is associated with high-grade prostate cancer (20). In addition, the mTOR pathway is also important in the regulation of sterol regulatory element-binding proteins (SREBPs), which are important transcription factors that control lipid and cholesterol homeostasis (21). A study reported that the intracellular accumulation of cholesterol lipid droplets is driven by loss of expression of the tumor-suppressor PTEN and subsequent activation of the PI3K–AKT–mTOR signaling pathway, which is also connected with high-grade prostate cancer in humans (22). The areas of cholesterol accumulation on the cell membrane are called lipid rafts, which could initiate downstream signaling pathways and lead to the growth and development of PCa. Statins could reduce the level of cholesterol and affect the formation of lipid rafts on the cell membrane, thereby affecting the androgen receptor (AR) pathway, epidermal growth factor receptor (EGFR) pathway, luteinizing hormone receptor pathway, and others (23–25), thus inhibiting downstream signaling pathways such as AKT and JAK-STAT3 (26), and then suppressing tumor cell growth and promoting cell apoptosis. Therefore, statins could affect the accumulation of cholesterol and block the necessary survival signals needed by tumors.

Additionally, cholesterol is the precursor of androgen, so statins can affect the synthesis of intracellular androgen by reducing the level of serum cholesterol, thereby affecting the growth of prostate cancer cells. An randomized controlled trial (RCT) showed that 80 mg/day of atorvastatin was associated with a reduction in serum androgen levels in PCa patients, but whether androgen levels in prostate tissue were also significantly reduced remains to be studied (27). Besides, statins could also suppress cancer cell proliferation by reducing the levels of mevalonate (MVA) and isoprenoids derived from it, such as farnesyl pyrophosphate (FPP) and geranylgeranyl pyrophosphate (GGPP), which were essential for posttranslational modifications of a variety of proteins called protein prenylation. Protein prenylation was important for the localization, membrane anchoring, and function of numerous signaling proteins, including Rho-GTPase family members such as Ras and the Rho GTPases, which could function as intermediators between extra- and intracellular signaling and regulate the activity of several kinases to regulate different physiological processes (28). Rho GTPases were tightly connected with growth-promoting pathways like mTOR and MAPK signaling pathways and contributed to tumorigenesis, metastasis, and drug resistance (29). Besides, the mevalonate pathway could influence Hippo/YAP signaling, which was important in tissue proliferation and tumorigenesis (30). Furthermore, statins could possibly induce apoptosis in cancer cells independent of their effect on cholesterol levels by suppressing cyclin−dependent kinase 2 (CDK2) or activating caspases and promoting cell-cycle arrest in PCa (31, 32).

More than 60 studies have reported the interaction between statin use and the prognosis of patients with PCa after definite treatment, including BCR, prostate cancer-specific mortality (PCSM), and overall survival (OS). The results of most literatures are encouraging but contradictory at the same time, which indicates that the effect of statins on the prognosis of PCa patients remains controversial. A meta-analysis of 34 observational studies published in 2016 showed that statin use could significantly reduce the risk of biochemical recurrence (BCR) in patients receiving RT (HR: 0.79, 95% CI: 0.65, 0.95, p = 0.01), but there was no statistically significant reduction in BCR risk in patients treated with RP (HR: 0.94, 95% CI: 0.81, 1.09, p = 0.43). Meanwhile, statins have a significant effect on the reduction of tumor metastasis, all-cause mortality, and PCSM after treatment (33). The investigators also observed a significant heterogeneity in the included studies. Since many new studies have been published since 2016, we decided to conduct this systematic review and meta-analysis to reevaluate the association between statin use and the risk of BCR among patients with prostate cancer after definite treatments.

## Materials and Methods

This systematic review and meta-analysis was conducted according to the Preferred Reporting Items for Systematic Reviews and Meta-analyses (PRISMA) 2020 reporting guideline (34).

### Criteria for Study Selection

All the studies were included into this systematic review and meta-analysis if they met the following criteria: (1) the exposure of interest was statin use; (2) the study design was cohort; (3) the outcome of interest was BCR of prostate cancer; (4) the follow-up ≥ 6 months; and (5) risk estimates and 95% confidence intervals (CIs) were reported (or information to calculate them). The animal studies, *in vitro* studies, RCTs, and case–control studies were excluded. No language or publication status limits were applied.

### Literature Search and Search Procedure

**Figure 1:**
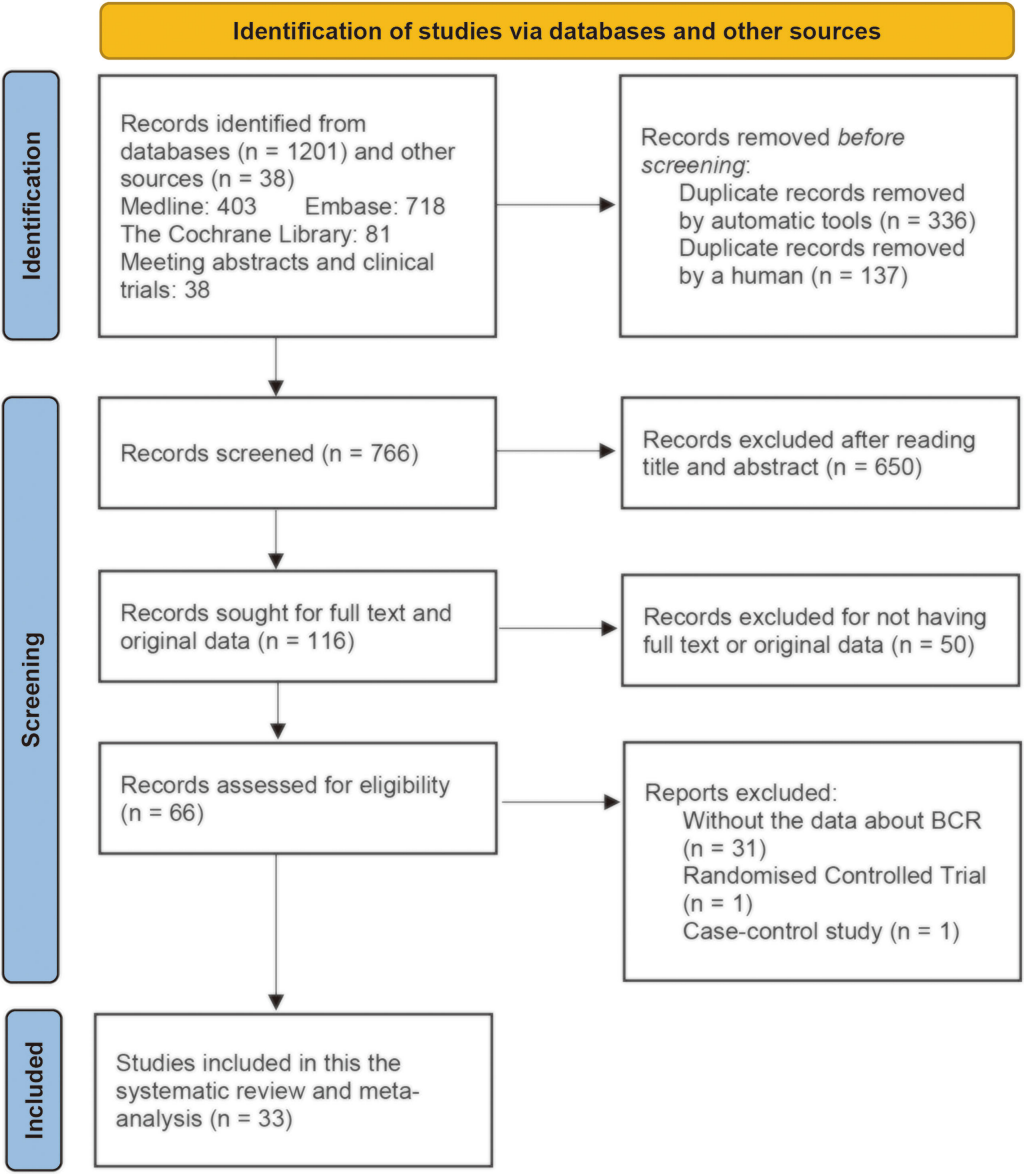
PRISMA (Preferred Reporting Items for Systematic Reviews and Meta-Analyses) flowchart for study selection for the systematic review on statins and clinical outcomes among patients with prostate cancer following definitive therapy.

We searched PubMed (Medline), EMBASE, and Cochrane Library for cohort studies evaluating the effects of statins on the BCR of patients with prostate cancer between January 1, 2000, and December 31, 2021. We also searched Google Scholar to retrieve gray literatures such as meeting abstracts. We searched these databases using key words such as “statins,” “HMG-CoA inhibitor,” “prostate cancer,” and “prostatic neoplasms.” The detailed search strategy for each database is reported in **Supplementary Table 1** with keywords and the number of retrieved citations per string. During the screening procedure, two reviewers (J-XS and X-YZ) independently searched abstracts and selected them according to the search criteria. The inter-rater kappa statistic was calculated to evaluate the consistency between the two authors for using the inclusion and exclusion criteria. Discrepancies about the inclusion or exclusion were resolved by consensus of the third author (Q-DX). The EndNote application (version X9) was used to remove the duplicates and apply the inclusion criteria. We utilized a PRISMA flowchart to depict the literature search procedure (**Figure 1**).

### Data Extraction

Three authors (J-XS, C-QL, and Q-DX) independently extracted information from the included studies using a designed data extraction sheet. The data extraction sheet consisted of bibliographic information and background information. Bibliographic information included author name, year of publication, and journal name and title. Background information included the inclusion and exclusion criteria for patients, age, follow-up period, body mass index (BMI), the level of serum cholesterol, race, the level of PSA, Gleason score (GS), tumor stage, primary treatment, the definition of statin use, the dose and median duration of usage of statins, definition of BCR, the number of patients, the number of statin users, and the number of patients with BCR. Moreover, we also extracted the data about the outcomes. The primary outcome of interest for this study was BCR. The adjusted multivariate hazard ratio (HR) and risk ratio (RR) with corresponding 95% confidence intervals (CIs) were used to assess the potential association between statin use and BCR after primary treatment.

### Literature Quality Assessment

We adapted the Newcastle–Ottawa scale (NOS) tool to assess the risk of bias of the included cohort studies. The NOS consists of three categories (Selection, Comparability, and Outcome) and a total of eight items (**Table 2**). A study can be awarded a maximum of one point for each numbered item within the Selection and Outcome categories, and a maximum of two points can be given for Comparability (68). Therefore, a study can be awarded at most nine points in total. The quality of the studies was considered as good, fair, or poor based on the Agency of Healthcare Research and Quality (AHRQ) standards using the scores obtained from the NOS (69).

### Data Synthesis and Analysis

We calculated a pooled hazard ratio (pHR) and a pooled risk ratio (pRR) with 95% confidence interval (CI) for BCR reported in the included studies using random effects (RE) models and quality effects (QE) models, respectively. We analyzed the heterogeneity between studies using the standard Cochrane chi-square χ^2^ (Cochrane’s Q) test with a significance level of α = 0.10 and the I^2^ test. An I^2^ statistic ≥50% indicates a considerable level of heterogeneity. The L’Abbé plot and Galbraith plot were used to visually display the heterogeneity of included studies. We performed subgroup analyses stratified by parameters such as primary treatment and country to find out the potential source of heterogeneity. We also performed meta-regression using parameters such as age, follow-up duration, publication year, PSA level, BMI (value or the percentage of BMI < 30 kg/m^2^), serum cholesterol level, percentage of patients in tumor stage ⩾T3, percentage of patients with Gleason score ⩾7, and percentage of patients with black race, which could be responsible for the differences in the outcomes observed among the studies. We determined the presence of publication bias in observational studies using both the Begg’s (70) and Egger’s (71) tests. A contour-enhanced funnel plot was utilized to determine other causes of publication bias by examining the symmetry of the plot. Further, we did sensitivity analyses and cumulative meta-analysis by stepwise adding or omitting included studies. We also applied the trim-and-fill method to evaluate the effect of publication bias (72), and a filled forest plot was constructed to preclude the publication bias on pHR and pRR. The meta-analyses using a QE model were performed using the MetaXL software to estimate the pHR and pRR. All the other data processing and statistical analysis were conducted by R software version 4.1.1. All the p-values were on two sides, and p-value <0.05 was considered with statistical significance.

## Results

A total of 1,239 publications were retrieved from electronic databases and gray literatures, and a total of 33 studies were selected and included in this systematic review and meta-analysis after employing exclusion criteria (**Figure 1**). A total of 473 duplicates were removed by automatic tools and artificial identification successively. A total of 650 records were excluded after reading the title and abstract, and 50 records were excluded for not having full-text or original data. After reading the full text, 31 records were excluded due to lack of data about BCR and two records were excluded because they did not belong to cohort studies (one RCT (73) and one case–control (74) study). Finally, 33 studies met the inclusion criteria for the current review. The inter-rater reliability between the two authors during the selection process was good (κ = 0.87).

### Characteristics of Included Studies

The characteristics of all the 33 studies are presented in **Table 1**. All the studies were observational cohort studies published between 2006 and 2021. Twenty-four studies were conducted in the United States (35, 37, 40, 43–48, 51–54, 56–59, 61–67), two in South Korea (42, 60), one in Portugal (38), one in Greece (50), one in France (55), two in Finland (36, 41), and one in Canada (39), and one study collected data from six centers located in the North America and Europe (49). The study cohort size ranged from 247 (43) to 6,842 (49) among the included studies. The percentage of statin users ranged from 11.4% (45) to 70.4% (43). All the included studies had at least 2 years of median or mean follow-up duration. The primary treatment of patients for 18 studies was RP, including open, laparoscopic, or robot-assisted RP. Nine studies used RT (either external beam, brachytherapy, or a combination of them) as primary treatment. Three studies included patients treated with RP or RT. Two studies chose ADT as their primary treatment, and one study brought into patients treated with RP or RT or ADT. In some studies, patients accepting adjuvant treatment before RP or RT were excluded (37, 45, 52, 53, 57, 60, 65). The definition of the BCR in most studies were the same: a posttreatment PSA value of 0.2 ng/ml or greater in men who underwent RP; nadir PSA level +2 ng/ml (Phoenix criteria), for men treated with RT; any PSA increase in men treated with primary ADT; and no evidence of clinical and/or radiographically detected disease. However, the definition of statin use was various in different studies. Patients were considered as statin users if they had ever used statins of any type or any dose at any time recorded in the medication database in many studies. However, the type, dose, or duration of statin use were strictly defined in some studies. For example, five studies recorded the dose of statin use (36, 40, 41, 50, 61) and four studies recorded the type of statins (42, 50, 61, 65). Three studies restricted the duration of statin use such as statin use longer than 10 years (35, 47, 51).

**Table 1:** Characteristics of included studies in the systematic review and meta-analysis.

Study	Year	Country	Follow-up period	Patient characteristics	Age (years), mean (SD) or median (IQR)	BMI (kg/m^2^), mean (SD) or median (IQR)	Cholesterol (mg/dL), mean (SD), or median (IQR)	Race	PSA (ng/mL), mean (SD), or median (IQR)	Gleason score	Tumor stage	Primary treatment (s)	Definition of statin use	Definition of BCR	No. of patients	No. of patients on statins (%)	Covariate adjustment	NOS
Nicole Prabhu et al. (35)	2021	USA	2002–2015, median 112.8 months (IQR 70.68–149.7)	Patients from the National Cancer Institute-funded Specialized Program of Research Excellence (SPORE)	(1) μ: 72.8 (7.28); (2) μ: 71.0 (8.07)	NR	NR	(1) 89.3% for white, 6.1% for AA; (2) 92.0% for white, 4.9% for AA	NR	(1) ≥7: 60.0%; (2) ≥7: 60.6%	(1) T0: 0.2%; T2b-2c: 59.3%; T3a–T4: 21.9%; (2) T0: 0.2%; T2b–2c: 59.6%; T3a–T4: 22.2%	RP	2 years prior to or any time subsequent to RP	A detectable or rising PSA greater than or equal to 0.2 ng/ml	3,088	1,222 (39.6%)	NR	8
A. I. Peltomaa et al. (36)	2021	Finland	1996–2015, average 6.3 years	Finnish randomized study of screening for prostate cancer in the metropolitan areas of Helsinki or Tampere	(1) μ: 69.7; (2) μ: 69.0	(1) M: 26.8 (24.7, 29.1); (2) M: 26.0 (23.7, 28.7)	NR	NR	(1) >20: 25.2%; (2) >20: 34.2%	(1) ≥7: 63.5%; (2) ≥7: 66.1%	(1) T1–T2: 70.0%; T3–T4: 30.0%; (2) T1–T2: 60.8%; T3–T4: 39.2%	ADT	After ADT initiation	Two consecutive rises of at least 50% from nadir PSA provided that the final PSA was over 2	4,428	2,544 (57.5%)	Adjusted by age, randomization group, medications, and PCa risk group	9
Linda My Huynh et al. (37)	2021	USA	2007–2020, mean 3.4 years (range 0.7–6.1)	Patients from University of California, Irvine, and University of Nebraska Medical Center. Patients undergoing adjuvant therapies following RP and/or those without follow-up were excluded	(1) μ: 64.7 (7.2); (2) μ: 61.9 (7.6)	(1) μ: 28.4 (4.5); (2) μ: 27.0 (3.8)	NR	NR	(1) μ: 8.5 (9.1); (2) μ: 10.3 (15.3)	(1) ≥7: 0%; (2) ≥7: 0%	(1) T1–T2: 95.7%; T3–T4: 4.3%; (2) T1–T2: 95.1%; T3–T4: 4.9%	RP or RT	Statins of any type	Two consecutive PSA values ≥ 0.2 ng/ml in the radical prostatectomy cohort and 2 ng/ml PSA above nadir in the radiation therapy cohorts, respectively	1,581	685 (43.3%)	NR	8
Roberto Jarimba et al. (38)	2020	Portugal	2009–2018, mean 51.2 months (range 19.9–82.5)	Patients from Centro Hospitalar e Universitário de Coimbra	(1) μ: 64.43 (6.60); (2) μ: 62.95 (6.96)	NR	NR	NR	(1) μ: 8.16 (5.45); (2) μ: 8.83 (8.9)	NR	(1) T2: 63.9%; T3: 35.7%; (2) T2: 65.7%; T3: 34.3%	RP	All hMG-CoA reductase inhibitors (including combination therapies such as ezetimibe/simvastatin); patients used statins at the date of surgery and did not suspend the drug afterward	Two consecutive PSA measurements >0.2 ng/ml after an undetectable PSA <0.1 ng/ml	702	400 (45.7%)	Adjusted for baseline demographic and clinical features	8
Viranda H. Jayalath et al. (39)	2018	Canada	1995–2016, median 50 months	Men at the Princess Margaret Cancer Center with low-risk prostate cancer at diagnosis (Gleason score <7, <4 positive cores, <50% involvement of any one core, and PSA <10.0 ng/dl), who had not undergone active treatment were eligible	(1) M: 65 (61–69); (2) M: 62 (57–67)	(1) <30: 76.5%; (2) <30: 80.8%	NR	(1) 83.4% for white, 6.2% for AA; (2) 81% for white, 6.9% for AA	(1) M: 4.9 (3.2, 6.5); (2) M: 4.9 (3.7, 6.4)	(1) ≥7: 0%; (2) ≥7: 0%	NR	RP or RT	A man was considered a statin user if one of the following criteria were fulfilled: (1) statin use was reported on the date of diagnosis; (2) dates for statin use encompassed the date of diagnosis; or (3) statin use was noted within 3 months after prostate cancer diagnosis	PSA ≥0.2 ng/ml after RP, a PSA ≥2 ng/ml above the nadir after RT, or the initiation of salvage therapy	291	67 (23.0%)	NR	8
Emma H. Allott et al. (40)	2018	USA	2008–2011, median 3.8 years	The North Carolina-Louisiana Prostate Cancer Project (PCaP)	(1) μ: 63.3 (7.0); (2) μ: 60.8 (7.8)	(1) <30: 53.3%; (2) <30: 66.8%	NR	(1) 58% for white, 42% for AA; (2) 53% for white, 47% for AA	(1) M: 5.2 (4.2–7.4); (2) M: 5.5 (4.3–8.3)	(1) ≥7: 16%; (2) ≥7: 16%	(1) T1: 61%; T2–T4: 39%; (2) T1: 63%; T2–T4: 37%	RP or RT	Research subjects gathered all prescription medications used in the 2-week period prior to interview and presented them to the research nurse at the time of interview for documentation of statin use	Undetectable PSA after RP that was followed by a PSA ≥0.2 ng/ml, confirmed with a second PSA ≥0.2 ng/ml or nadir (lowest PSA achieved after radiation) + 2 ng/ml	669	244 (36.5%)	Adjusted for age, race, and obesity status	7
Teemu Keskivali et al. (41)	2016	Finland	1995–2009, median 8.6 years	Men who accepted prostate cancer treatment at the Tampere University Hospital (TAUH)	(1) M: 63; (2) M: 63	NR	(1) M: 206.4 (185.6, 235.9); (2) M: 193.3 (174.0, 216.6)	NR	(1) ≤4.8: 49.4%; (2) ≤4.8: 50.5%	(1) ≥7: 52.5%; (2) ≥7: 56.6%	(1) T1–2 N0/x M0/x: 99.2%; T3 and/or N1 and/or M1: 0.8%; (2) T1–2 N0/x M0/x: 98.4%; T3 and/or N1 and/or M1: 1.6%	RP	Any statin use recorded in the Finnish national prescription database	Two consecutive PSA values of 0.2 ng/ml or above or radiological progression after prostatectomy	1,314	528 (40.2%)	Adjusted for age at surgery, tumor stage and Gleason grade, PSA level at the time of diagnosis, surgical margin positivity, total cholesterol, and use of antidiabetic and antihypertensive drug	8
Cheryn Song et al. (42)	2015	South Korea	1998–2011, median 32 months (IQR: 18.2, 55.2)	Korean patients with prostate cancer who had undergone RP at Asan Medical Center	(1) M: 67 (63, 70); (2) M: 67 (63, 71)	(1) M: 25.2 (23.4, 27.5); (2) M: 24.4 (22.6, 26.4)	(1) M: 179 (159, 201); (2) M: 166 (143, 199)	NR	(1) M: 6.2 (4.5, 9.5); (2) M: 6.9 (4.7, 11.2)	(1) ≥7: 61.4%; (2) ≥7: 53.7%	(1) T2: 74.8%; T3: 24%; N+: 2%; (2) T2: 64.5%; T3a: 32%; N+: 3%	RP	Preoperative statin use from each patient’s medical record; postoperative statin use was evaluated through telephone survey	PSA greater than 0.2 ng/ml	2137	452 (21.2%)	NR	7
Daniel S. Oh et al. (43)	2015	USA	1999–2009, 51 months (range 9.4–140.35)	Men with prostate cancer treated at the Durham Veterans affairs Medical center	(1) μ: 62.8; (2) μ: 61.4	NR	NR	NR	(1) >10: 5.7%; (2) >10: 15.0%	(1) ≥7: 8.1%; (2) ≥7: 17.8%	(1) T1–T2a: 97%; T2b–c: 3%; T3–4: 0.0%; (2) T1–T2a: 92%; T2b–c: 7%; T3–4: 1%	RT	Patients were identified as statin users or non-statin users either at time of consultation or during follow-up	Rise of 2 ng/ml or more above the nadir after RT	247	174 (70.4%)	Adjusted for clinical T stage, Gleason score, PSA, and brachytherapy characteristics	7
John Cuaron et al. (44)	2015	USA	1998–2010, 48 months (range 1–156)	Patients with clinically localized prostate cancer at Memorial Sloan Kettering Cancer Center	(1) ≥65: 74%; (2) ≥65: 73%	NR	NR	NR	(1) >10:18%; (2) >10: 29%	(1) ≥7: 84%; (2) ≥7: 85%	1) T1–T2a: 79%; T2b–T2c: 18%; T3a+: 3%; (2) T1–T2a: 75%; T2b–T2c: 19%; T3a+: 6%	RT	Take a statin medication (statin group) before initiating RT	Phoenix nadir + 2 definition	754	273 (36.2%)	NR	7
MR Danzig et al. (45)	2015	USA	1995–2012, median 27 months	Diabetic patients in Columbia Urologic Oncology database, those accepting adjuvant radiation or hormonal therapy were excluded	(1) M: 65; (2) M: 63	NR	NR	(1) 31.6% for white, 26.3% for AA; (2) 36.4% for white, 23.9% for AA	(1) M: 5.72; (2) M: 6.05	(1) ≥7: 65%; (2) ≥7: 54%	(1) T1–T2: 98.0%; T3–T4: 2.0%; (2) T1–T2: 96.5%; T3–T4: 3.5%	RP	record in the database	The first recorded PSA of more than 0.2 ng/ml	669	76 (11.4%)	NR	6
Lauren C. Harshman et al. (46)	2015	USA	1996–2013, median 5.8 years (IQR: 0.1–15.9)	Patients with hormone-sensitive PC from Dana-Farber Cancer Institute	(1) M: 62 (56, 67); (2) M: 60 (55, 66)	NR	NR	(1) 93% for white, 4% for AA; (2) 93% for white, 5% for AA	(1) M: 9.1 (6, 17); (2) M: 11.8 (6, 40)	(1) ≥7: 73%; (2) ≥7: 74%	(1) T1–T2: 76%; T3-T4: 4%; (2) T1–T2: 67%; T3–T4: 6%	ADT	Patients were defined as statin users if they were using statins at the time of ADT initiation	A minimum of 2 increases in PSA level	926	283 (30.6%)	Adjusted for predefined prognostic clinical factors including biopsy Gleason score, type of primary therapy, use of prior ADT in conjunction with localized therapy, metastatic status, and PSA level at initiation of ADT	7
Miriam B. Ishak-Howard et al. (47)	2014	USA	1999–2009, mean 94.9 months (SD = 56.6)	Study subjects came from the University of Michigan Prostate Cancer Genetic Project (PCGP)	(1) μ: 58.0 (7.4); (2) μ: 55.2 (7.6)	(1) <30: 82.1%; (2) <30: 85.8%	NR	(1) 96.5% for white, 2.5% for AA; (2) 97.5% for white, 2.1% for AA	(1) μ: 7.9 (10.2); (2) μ: 7.1 (9.1)	(1) ≥7: 50.4%; (2) ≥7: 51.3%	(1) T2: 65.9%; T3: 20.9%; (2) T2: 70.4%; T3: 21.0%	RP	Any statin use over the last 10 years	A single PSA test value of ≥0.4 ng/ml following an undetectable PSA (<0.1 ng/ml) after RP	539	258 (47.9%)	Adjusted for age at time of surgery, BMI, NSAID use, Gleason grade, pre-diagnostic PSA, clinical stage, and decade of surgery	7
Emma H. Allott et al. (48)	2014	USA	1996–2009, median 76.2 months (IQR: 45.1–108.8)	Patients undergoing RP in Shared Equal Access Regional Cancer Hospital (SEARCH) Database*^a^*, not including patients treated with preoperative ADT or RT	(1) μ: 60.6 (6.3); (2) μ: 60.7 (6.5)	(1) M: 27.6 (25.1–30.3); (2) M: 27.1 (24.3–30.1)	(1) M: 202 (181–224); (2) M: 185 (165–208)	(1) 51% for white, 42% for AA; (2) 51% for white, 45% for AA	(1) M: 5.9 (4.7, 9.1); (2) M: 7.1 (5.1, 10.7)	(1) ≥7: 29%; (2) ≥7: 40%	(1) T1: 61%; T2/T3: 39%; (2) T1: 64%; T2/T3: 36%	RP	Postoperative statin use	A single PSA >0.2 ng/ml, two consecutive concentrations at 0.2 ng/ml, or secondary treatment for detectable postoperative PSA	1,146	400 (34.9%)	Adjusted for age, race, PSA, BMI, pathological Gleason score, year of surgery, positive surgical margins, extracapsular extension, seminal vesicle invasion, lymph node, involvement and center	7
M Rieken et al. (49)	2013	Multi-countries*^b^*	2000–2011, median 25 months (IQR: 8–42)	patients with clinically localized PC treated with RP from six North American and European centers, not including patients treated with preoperative RT, hormonal treatment or chemotherapy	(1) μ: 61.7 (6.5); (2) μ: 61.0 (6.7)	NR	NR	NR	(1) μ: 7.7 (5.5); (2) μ: 7.5 (6.0)	(1) ≥7: 43.5%; (2) ≥7: 45.8%	(1) T3: 25.3%; N1: 10.9%; (2) T3: 25.5%; N1: 11.4%	RP	Statin use at the time of diagnosis, regardless of statin type, dose, or cumulative exposure	PSA value >0.2 ng/mL on two consecutive visits	6,842	2,275 (33.3%)	Adjusted for statin use, age (continuous), preoperative PSA (continuous), RP Gleason score, positive lymph nodes, positive surgical margins, stage pT3a, stage T3b	9
M. Kontraros et al. (50)	2013	Greece	1999–2010, mean 3.6 years (SD = 2.6), median 3.4 years (IQR: 1.5–5.0)	Patients without any antiandrogen or 5ARI medication preoperatively from Sismanoglio Hospital of Attiki and Gennimatas General Hospital of Athens	(1) μ: 65.4 (5.2); (2) μ: 65.2 (5.8)	(1) <25: 20.6%; (2) <25: 26.0%	NR	NR	(1) M: 7.2 (5.6, 9.7); (2) M: 8.0 (6, 10.6)	(1) ≥7: 55.1%; (2) ≥7: 46.7%	(1) cT1c: 70.1%; cT2: 29.9%; (2) cT1c: 69.6%; cT2: 30.4%	RP	Any statin use preoperatively or postoperatively	NR	588	170 (28.9%)	Adjusted for preoperative serum PSA, Gleason score more than seven, stage, positive surgical margins, and statin use	6
Milan S. Geybels et al. (51)	2013	USA	2002–2005, average 6.1 years	PCa patients aged 35–74 at diagnosis from a population-based, case–control study of PCa *via* the SEER Program cancer registry	(1) μ: 63.1 (6.8); (2) μ: 60.9 (8.1)	(1) μ: 27.0 (4.0); (2) μ: 28.5 (4.5)	NR	(1) White: 87.5%; (2) white: 82.9%	(1) M: 5.7 (4.4, 8.5); (2) M: 6.3 (4.7, 9.5)	(1) ≥7: 47.1%; (2) ≥7: 47.1%	(1) Local: 72%; (2) Local: 73%	RP or RT or ADT	Users were defined as men who reported having taken a statin at least once a week for 3 months or longer	A posttreatment PSA value of 0.2 ng/ml or greater in men who underwent RP; nadir PSA level +2 ng/ml (Phoenix criteria), for men treated with RT; or any PSA increase in men treated with primary ADT	685	208 (30.4%)	Adjusted for age at diagnosis (years), Gleason score, stage at diagnosis, diagnostic PSA level, primary treatment approach, race, first-degree family history of PCa, body mass index, smoking status, lifetime alcohol consumption, aspirin use, non-aspirin NSAID use, history of diabetes mellitus, and history of PCa screening	7
Chun Chao et al. (52)	2013	USA	2004–2011, mean 4.1 years (SD = 1.4)	Patients ≥40 years who were diagnosed with incident prostate cancer in Kaiser Permanente Southern California, those with stage IV disease or unknown stage and received RP prior to RT were excluded	(1) μ: 69.3 (5.9); (2) μ: 67.5 (8.0)	(1) <30: 71.8%; (2) <30: 85.2%	NR	(1) 63.6% for white, 19.0% for AA; (2) 59.0% for white, 19.0% for AA	(1) μ: 6.2 (6.7); (2) μ: 6.7 (7.8)	(1) ≥7: 52.1%; (2) ≥7: 49.6%	(1) Stage II: 97.5%; stage III: 2.5%; (2) Stage II: 98.7%; stage III: 1.3%	RT	Statin use prior to RT procedures	A rise in PSA by 2 ng/ml or more above the nadir PSA after radiation therapy based on the 2005 Phoenix definition	774	401 (51.8%)	Adjusted for race, stage, Gleason score, pre-radiotherapy PSA (continuous), hypertension, use of neoadjuvant therapy, and time from prostate cancer diagnosis to RT (continuous)	7
Chun Chao et al. (53)	2013	USA	2004–2010, mean 4.3 years (SD = 1.3)	All men aged 40 years and older with incident prostate cancer in the Kaiser Permanente Southern California (KPSC); those with stage IV disease or received neoadjuvant therapy prior to RP were excluded	(1) μ: 61.0 (6.0); (2) μ: 59.0 (7.0)	(1) <30: 77%; (2) <30: 85%	NR	(1) 59% for white, 17% for AA; (2) 64% for white, 16% for AA	(1) μ: 6.7 (4.2); (2) μ: 7.1 (6.4)	(1) ≥7: 48%; (2) ≥7: 44%	(1) Stage II: 85%; stage III: 15%; (2) Stage II: 87%; stage III: 13%	RP	Statin use prior to prostatectomy from KPSC’s electronic pharmacy records	A single PSA level >0.2 ng/ml after an undetectable PSA measurement (<0.1 ng/ml) after surgery	1,184	446 (37.7%)	Adjusted for age, race, stage, Gleason score, preoperative PSA, time from prostate cancer diagnosis to surgery, and obesity	7
Alon Y. Mass et al. (54)	2012	USA	2000–2008, median 57 months	Patients with clinically localized PCa at New York University	(1) μ: 58.96 (6.68); (2) μ: 58.17 (6.98)	(1) <30: 69.8%; (2) <30: 68.8%	NR	(1) 90.6% for white, 3.4% for AA; (2) 90.5% for white, 4.1% for AA	(1) M: 5.1 (4.0, 6.8); (2) M: 5.0 (4.0, 7.0)	(1) ≥7: 42.1%; (2) ≥7: 34.6%	(1) T1: 82.0%; T2–T3a: 18.0%; (2) T1: 81.6%; T2–T3a: 18.4%	RP	Statin medication use (ever vs. never) was extracted from patient medical records	PSA greater than 0.2 ng/ml with a confirmatory reading above this threshold	1,446	437 (30.2%)	Adjusted for age at diagnosis, preoperative PSA, pathological tumor stage, postoperative pathological Gleason score, and race	8
V. Misrai et al. (55)	2012	France	2004–2008, mean 33 months (SD = 10)	NR	(1) M: 64 (61, 70); (2) M: 64 (59, 60)	(1) <30: 43.3%; (2) <30: 51.8%	NR	NR	(1) M: 6.6 (4.8, 8.1); (2) M: 6.4 (4.7, 8.6)	(1) ≥7: 72.1%; (2) ≥7: 77.1%	(1) T1c: 67%; T2: 31%; T3: 2%; (2) T1c: 71%; T2: 26%; T3: 1%	RP	Collect the dose and type of statins from anesthesia records	An elevation of PSA >0.2 ng/ml on two successive dosages postoperatively	377	97 (25.7%)	Adjustment for the D’Amico group criterion and the other confounding factors (type 2 diabetes and positive surgical margins)	6
Nicholas G Zaorsky et al. (56)	2012	USA	1986–2006, median 75 months (range: 18–239)	Men with clinical stage T1–4, N0/X, M0 adenocarcinoma of the prostate without ADT	M: 69 (36, 86)	NR	NR	NR	>10: 30%	≥7: 26%	T1: 58%; T2: 39%; T3: 3%	RT	Stain drugs included atorvastatin, fluvastatin, lovastatin, pitavastatin, pravastatin, rosuvastatin, and simvastatin	Nadir + 2 ng/ml	2,045	689 (33.7%)	NR	7
Chad R. Ritch et al. (57)	2011	USA	1990–2008, median 36 months	Patients from the Columbia University Comprehensive Urologic Oncology Database, and patients were excluded from the analysis if they had (1) <2 years of adequate follow-up, (2) neo-adjuvant or adjuvant therapy in the form of hormones, radiation and/or chemotherapy, and (3) insufficient pathological data	(1) μ: 62; (2) μ: 59	NR	NR	(1) 63.7% for white, 14.6% for AA; (2) 71.1% for white, 11.8% for AA	(1) M: 6.4; (2) M: 7.1	(1) M: 6.4; (2) M: 7.1	(1) T1: 92.5%; T2–T3: 7.5%; (2) T1: 86.4%; T2–T3: 13.6%	RP	Data on statin use were extracted from the admission or discharge records of patients at the time of RP	PSA ≥0.2 ng/ml after a previously undetectable PSA 3 months postoperatively	1261	281 (22.3%)	NR	7
Alison M. Mondul et al. (58)	2011	USA	1993–2006, median 7 years	Patients with clinically localized prostate cancer at the Johns Hopkins Hospital, those who received hormone or RT before prostatectomy were excluded	(1) μ: 57.7; (2) μ: 56.0	(1) μ: 26.7; (2) μ: 26.3	NR	(1) 93% for white, 1.4% for AA; (2) 92.4% for white, 3% for AA	(1) μ: 6.3; (2) μ: 7.1	(1) ≥7: 19.7%; (2) ≥7: 17.9%	(1) T1: 73.7%; T2–T3a: 26.3% (2) T1: 67.7%; T2–T3a: 32.1%	RP	Statin use starting before or after surgery	A confirmed repeat PSA increase from a nadir of nondetectable to 0.2 ng/ml or greater	1,583	779 (49.2%)	Adjusted for age, race, BMI, smoking, prostate cancer family history, aspirin use, and ACE inhibitor use at prostatectomy, surgery calendar year, preoperative PSA, pathological stage, and Gleason sum	8
Marisa A Kollmeier et al. (59)	2011	USA	1995–2007, median 5.9 years (range, 0–14 years; IQR: 3.5–10.5 years)	Patients treated at Memorial Sloan-Kettering Cancer Center for clinically localized stage T1–T3 prostatic adenocarcinoma	(1) ≥65: 74%; (2) ≥65: 73%	NR	NR	NR	(1) >10: 27%; (2) >10: 39%	(1) ≥7: 52%; (2) ≥7: 55%	(1) T1: 56%; T2: 37%; T3: 7%; (2) T1: 48%; T2: 40%; T3: 12%	RT	All HMG-CoA reductase inhibitor according to medical record review	Nadir +2 definition	1,681	382 (22.7%)	NR	7
JH Ku et al. (60)	2011	South Korea	1997–2009, median 38.0 months (range: 3–143)	Patients who underwent retropubic RP and who did not receive neoadjuvant treatment at Seoul National University Hospital	(1) μ: 65.3 (6.8) (2) μ: 65.2 (6.7)	(1) <30: 97.6%; (2) <30: 99.2%	NR	NR	(1) μ: 9.6 (9.3); (2) μ: 13.6 (20.5)	(1) ≥7: 59.8%; (2) ≥7: 52.5%	(1) T1: 59.8%; T2–T3: 40.2%; (2) T1: 59.7%; T2–T3: 40.4%	RP	All 3-hydroxy-3-methyl-glutaryl-co-enzyme A reductase inhibitors	A single PSA of 0.2 ng/ml or greater with another increasing value	609	79 (13.0%)	NR	8
Robert J. Hamilton et al. (61)	2010	USA	1988–2008, median 38 months (IQR: 13–68) for non-statin users, median 24 months (IQR: 11–52) for statin users	Men treated with RP from the Shared Equal Access Regional Cancer Hospital (SEARCH) Database	(1) μ: 62.6 (5.6); (2) μ: 60.6 (6.6)	(1) <30: 59.7%; (2) <30: 69.2%	NR	(1) 53% for white, 36% for AA; (2) 48% for white, 47% for AA	(1) M: 6.2 (4.7, 9.1); (2) M: 6.9 (4.9, 10.5)	(1) ≥7: 50%; (2) ≥7: 38%	(1) T1c: 67%; T2/T3: 33%; (2) T1c: 58%; T2/T3: 42%	RP	Statin use at surgery	A single PSA >0.2 ng/ml, 2 concentrations at 0.2 ng/ml, or secondary treatment for detectable postoperative PSA	1,319	236 (17.9%)	Adjusted for clinical and pathological characteristics: pathological Gleason score, extracapsular extension, seminal vesicle invasion, positive surgical margins, and lymph node metastases	7
L. Spencer Krane et al. (62)	2010	USA	2001–2008, mean 26 months	Men with biopsy proven prostate cancer at the Vattikuti Urology Institute	(1) μ: 61.4 (6.6); (2) μ: 59.4 (7.5)	(1) M: 28 (26, 30), <30: 67.7%; (2) M: 27 (25, 30), <30: 72.1%	NR	NR	(1) M: 5.0 (4.1, 6.5); (2) M: 5.2 (4.1, 7.2)	(1) ≥7: 69%; (2) ≥7: 64%	(1) T1c: 73; T2/T3: 27%; (2) T1c: 73; T2/T3: 27%	RP	All hMG-CoA reductase inhibitors (including combination therapies such as ezetimibe/simvastatin)	Single PSA of 0.2 ng/ml or greater with another increasing value	3,828	1,031 (26.9%)	NR	7
Jorge Rioja et al. (63)	2010	USA	2003–2009	Patients were treated at Memorial Sloan-Kettering Cancer Center	(1) M: 62 (57, 66); (2) M: 59 (54, 64)	NR	NR	NR	(1) M: 5.1 (3.8, 7.0); (2) M: 5.3 (3.9, 7.5)	(1) ≥7: 75%; (2) ≥7: 71%	NR	RP	We ascertained statin use from a prospective database	NR	3,748	1,084 (27.7%)	NR	7
Ruchika Gutt et al. (64)	2010	USA	1988–2006, median 50 months	Patients were treated at the University of Chicago Pritzker School of Medicine for nonmetastatic prostate adenocarcinoma, those with prior prostatectomy were excluded	(1) M: 69 (42, 83); (2) M: 68 (44, 83)	NR	(1) M: 186 (104, 315); (2) M: 192 (92, 292)	(1) 48% for white, 49% for AA; (2) 42% for white, 53% for AA	(1) >10: 34%; (2) >10: 43%	(1) ≥7: 54%; (2) ≥7: 37%	(1) T1–2a: 88%; T2b–c: 9%; T3-4: 3%; (2) T1–2a: 79%; T2b–c: 15%; T3–4: 7%	RT	Statin therapy during RT or during follow-up	The Phoenix definition (PSA nadir + 2 ng/ml)	691	189 (27.4%)	NR	8
Daniel E. Soto et al. (65)	2009	USA	1987–2006, median 47 months (range 2.5 months to 16.5 years)	Patients with localized prostate cancer who were treated at the University of Michigan Cancer Center, exclusion criteria included the presence of known lymphatic metastases, nonpelvic metastatic disease, the use of neoadjuvant or adjuvant chemotherapy, and a history of prostatectomy, cryosurgery, or brachytherapy	(1) μ: 68.0 (7.2); (2) μ: 68.2 (7.3)	NR	NR	(1) 90.9% for white, 8.4% for AA; (2) 90.8% for white, 8.4% for AA	(1) M: 3.1 (0.2, 12.0); (2) M: 4.6 (0.2, 17.2)	(1) ≥7: 58.9% (2) ≥7: 56.5%	(1) T1: 55%; T2: 42%; T3: 3%; (2) T1: 37%; T2: 51%; T3: 12%	RT	Statin use before the start of RT	Phoenix definition of a current PSA nadir + 2 ng/ml or the initiation of salvage ADT	968	220 (22.7%)	The total radiation dose, T stage, iPSA level, ADT use, pelvic RT use, and year of treatment	7
A. M. Shippy et al. (66)	2007	USA	1995–2000, median 85 months	Men with clinical stage T1–3 prostate adenocarcinoma at Memorial Sloan-Kettering Cancer Center	NR	NR	NR	NR	NR	NR	NR	RT	NR	According to the nadir + 2 definition	871	168 (19.3%)	NR	4
N. K. Sharma et al. (67)	2006	USA	1995–2000, median 58.1 months	Patients from Fox Chase Cancer Center; those who initially treated with ADT were excluded	M: 69 (43, 84)	NR	NR	NR	M: 7.4	≥7: 31%	T1–T2: 90%; T3–T4: 3.5%	RT	Statin use before, during and after RT	Using the ASTRO (3 rises) and Nadir 2 ng/ml (Phoenix) criteria	983	178 (18.1%)	Age (continuous), dose (continuous), Gleason score, iPSA (continuous), stage	6

RT, radiation therapy; RP, radical prostatectomy; ADT, androgen deprivation therapy; BCR, biochemical recurrence; BMI, body mass index; IQR, interquartile range; SD, standard deviation; USA, United States of America; AA: African American; NOS, Newcastle–Ottawa scale; NR, not reported.

(1) denotes statin users and (2) non-statin users.

^a^Including five Veterans Administration (VA) Medical Centers (Palo Alto, CA; West Los Angeles, CA; Durham, NC; Asheville, NC; Augusta, GA).

^b^Including Department of Urology, Weill Cornell Medical College, New York Presbyterian Hospital, New York, NY, USA; Department of Urology, University of Montreal, Montreal, QC, Canada; Department of Urology, University of Texas Southwestern Medical Center, Dallas, TX, USA; Department of Urology, Urological Research Institute, San Raffaele Scientific Institute, Milan, Italy; Prostate Cancer Center, Hospital Barmherzige Schwestern Linz, Linz, Austria; Department of Urology, Medical University of Graz, Graz, Austria.

**Table 2:** Newcastle–Ottawa Scale for assessing the quality of studies in meta-analysis.

Study	Selection				Comparability	Outcome			Score
	Representativeness of the exposed cohort	Selection of the non-exposed cohort	Ascertainment of exposure	Demonstration that outcome of interest was not present at start of the study	Comparability of cohorts on the basis of the design or analysis	Assessment of outcome	Was followed up long enough for outcomes to occur	Adequacy of follow-up of cohorts	
Nicole Prabhu et al, (35)	☆	☆	☆	☆	☆	☆	☆	☆	8
A. I. Peltomaa et al, (36)	☆	☆	☆	☆	☆☆	☆	☆	☆	9
Linda My Huynh et al, (37)	☆	☆	☆	☆	☆	☆	☆	☆	8
Roberto Jarimba et al, (38)	☆	☆	☆	☆	☆	☆	☆	☆	8
Viranda H. Jayalath et al, (39)	☆	☆	☆	☆	☆	☆	☆	☆	8
Emma H. Allott et al, (40)	☆	☆	☆	☆		☆	☆	☆	7
Teemu Keskivali et al, (41)	☆	☆	☆	☆	☆	☆	☆	☆	8
Cheryn Song et al, (42)	☆	☆	☆	☆		☆	☆	☆	7
Daniel S. Oh et al, (43)	☆	☆	☆	☆		☆	☆	☆	7
John Cuaron et al, (44)	☆	☆	☆	☆		☆	☆	☆	7
MR Danzig et al, (45)		☆	☆	☆		☆	☆	☆	6
Lauren C. Harshman et al, (46)	☆	☆	☆	☆		☆	☆	☆	7
Miriam B. Ishak-Howard et al, (47)	☆	☆	☆	☆		☆	☆	☆	7
Emma H. Allott et al, (75)	☆	☆	☆	☆		☆	☆	☆	7
M Rieken et al, (49)	☆	☆	☆	☆	☆☆	☆	☆	☆	9
M. Kontraros et al, (50)	☆	☆	☆	☆		☆	☆		6
Milan S. Geybels et al, (51)	☆	☆	☆	☆		☆	☆	☆	7
Chun Chao et al, (52) (RT)	☆	☆	☆	☆		☆	☆	☆	7
Chun Chao et al, (53) (RP)	☆	☆	☆	☆		☆	☆	☆	7
Alon Y. Mass et al, (54)	☆	☆	☆	☆	☆	☆	☆	☆	8
V. Misrai et al, (55)			☆	☆	☆	☆	☆	☆	6
Nicholas G Zaorsky et al, (56)	☆	☆	☆	☆		☆	☆	☆	7
Chad R. Ritch et al, (57)	☆	☆	☆	☆		☆	☆	☆	7
Alison M. Mondul et al, (58)	☆	☆	☆	☆	☆	☆	☆	☆	8
Marisa A Kollmeier et al, (59)	☆	☆	☆	☆		☆	☆	☆	7
JH Ku et al, (60)	☆	☆	☆	☆	☆	☆	☆	☆	8
Robert J. Hamilton et al, (61)	☆	☆	☆	☆		☆	☆	☆	7
L. Spencer Krane et al, (62)	☆	☆	☆	☆		☆	☆	☆	7
Jorge Rioja et al, (63)	☆	☆	☆	☆	☆	☆	☆	☆	7
Ruchika Gutt et al, (64)	☆	☆	☆	☆	☆	☆	☆	☆	8
Daniel E. Soto et al, (65)	☆	☆	☆	☆		☆	☆	☆	7
A. M. Shippy et al, (66)	☆	☆		☆		☆			4
N. K. Sharma et al, (67)	☆	☆	☆	☆		☆	☆		6

A study can be awarded a maximum of one star for each numbered item within the Selection and Outcome categories. A maximum of two stars can be given for Comparability.

### Characteristics of Patients With Prostate Cancer

The mean or median age of the patients in the included studies ranged from 55.2 to 72.8 years. Seventeen studies collected the data about BMI, in the form of either BMI value or the percentage of patients with BMI < 30 kg/m^2^. The mean or median BMI of patients ranged from 24.4 to 28.4, and the percentage of patients with BMI < 30 kg/m^2^ ranged from 43.4% to 99.2%, which indicated that most patients included were overweight or obese. Only four studies provided the data about the level of serum cholesterol (41, 42, 48, 64). The median serum cholesterol level ranged from 166 to 206.4 mg/dl. The majority of studies consist of mainly patients of white race with black race less than 20%. However, the patients with black race constituted >30% in five studies (40, 45, 48, 61, 64). There were no significant differences in race and statin use. Thirty-one studies reported the level of PSA before primary treatment. The median or mean PSA level for statin users ranged from 3.1 to 9.6 ng/ml, and the percentage of patients with PSA > 10 ng/ml ranged from 5.7% to 30%. Statin users had a significant lower baseline PSA level than non-users. Thirty-one studies reported the Gleason score, and the percentage of patients with Gleason score ≥7 in the majority of studies was between 50% and 70%. However, all the patients had a Gleason score <7 in two studies (37, 39). There were no significant differences in Gleason score and statin use. Twenty-nine studies recorded the clinical tumor stage of prostate cancer. The tumor stage varies dramatically in different studies, and there existed a significant difference between tumor stage among statin users and non-users in some studies.

### Quality Assessment of the Included Studies

We applied the NOS tool to assess the quality of included studies (**Table 1**). The majority of the included studies had a good or fair quality except two meeting abstracts (66, 67) for lack of complete data.

### Statin Use and the HR and RR of BCR

**Figure 2:**
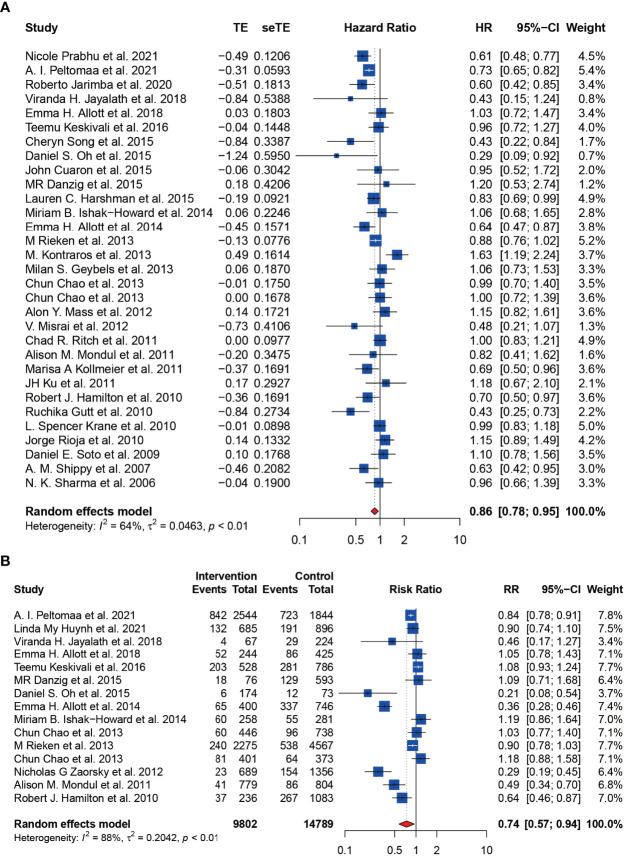
The effect of statins on BCR risk of prostate cancer among men following definitive therapy using the random effects model. **(A)** Forest plot for the HR of BCR. **(B)** Forest plot for the RR of BCR.

**Figure 3:**
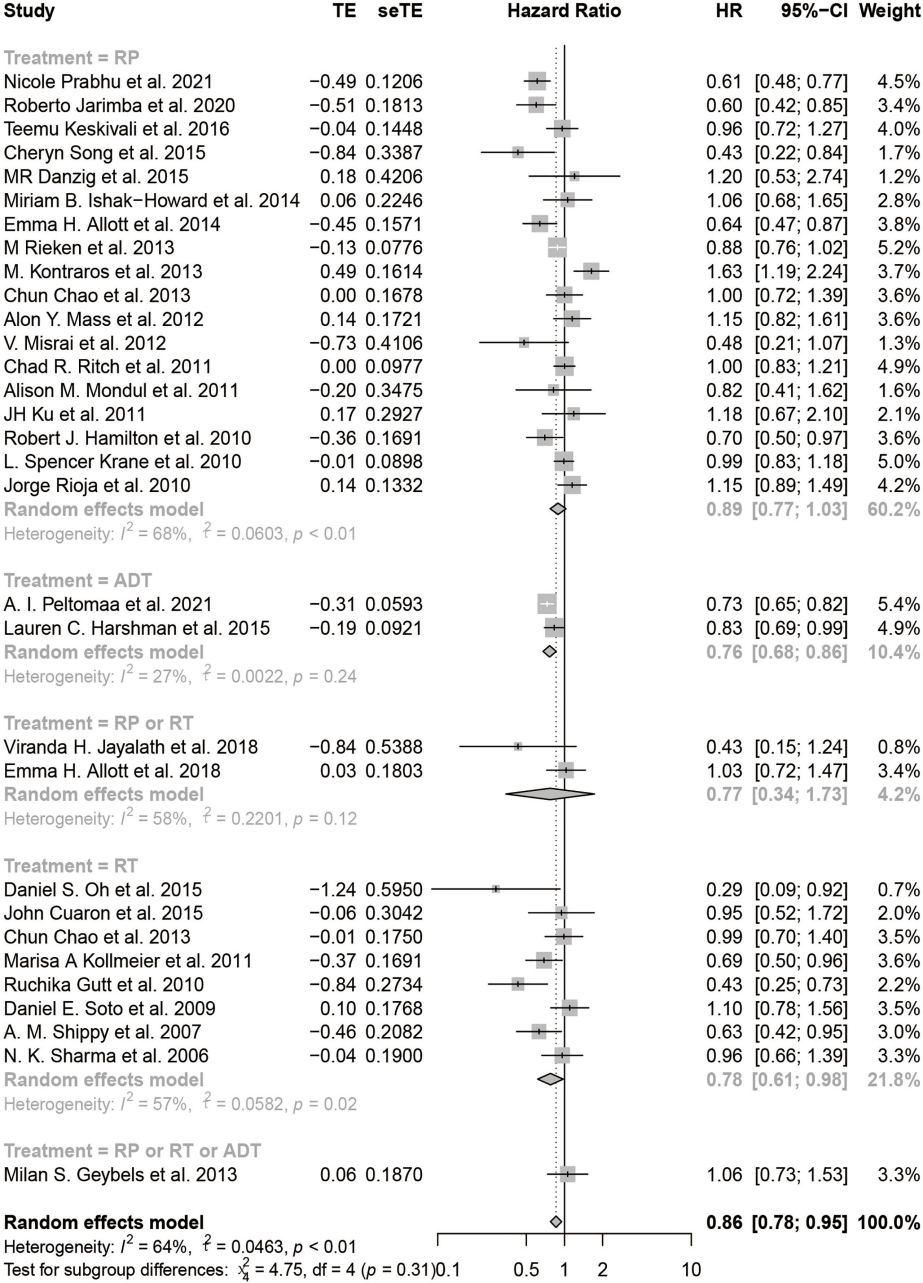
The forest plot for the HR of BCR with subgroup analyses by primary treatment.

The HR of BCR was reported in 31 including studies, and the RR of BCR was reported in 15 including studies. As shown in the forest plots, statin users were significantly less likely to experience the BCR of prostate cancer after primary treatment, with a pHR of 0.86 (95% CI: 0.78 to 0.95, I^2^ = 64%, random effects model, **Figure 2A**) and a pRR of 0.74 (95% CI: 0.57 to 0.94, 24591 patients, I^2^ = 88%, random effects model, **Figure 2B**). Subgroup analyses according to primary treatment for HR showed that there still existed significant BCR reduction among patients accepting RT (pHR: 0.78, 95% CI: 0.61 to 0.98, I^2^ = 57%, random effects model, **Figure 3**) or ADT (pHR: 0.76, 95% CI: 0.68 to 0.86, I^2^ = 27%, random effects model, **Figure 3**) as their primary treatment, which was consistent with previously published articles (33, 76). However, as for RR, the subgroup analyses according to primary treatment exhibited that there just existed a significant difference in patients accepting ADT but only one study was divided into this group (**Figure S1A**). Subgroup analyses according to country for both RR (**Figure S1B**) and HR (**Figure S1C**) showed that statin use was significantly associated with BCR reduction in patients from USA.

**Figure 4:**
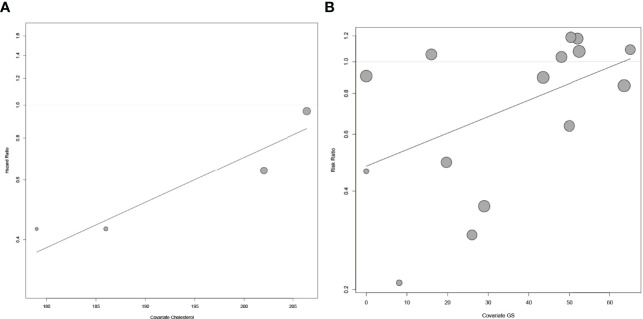
The meta-regression for risk of BCR and covariates. **(A)** The meta-regression for HR of BCR and the level of serum cholesterol. **(B)** The meta-regression for RR of BCR and GS. Each dot represents an individual study. Symbol size represents sample size.

As the heterogeneity was high in both the main analysis and subgroup analyses, we then performed meta-regression to find out the covariates causing this variability. We used publication year, follow-up duration, age, BMI value, the percentage of BMI <30 kg/m^2^, serum cholesterol level, the percentage of AA, serum PSA level, GS, and stage to construct the univariate meta-regression model. For HR, we found that the serum cholesterol level was significantly associated with BCR (p = 0.0074, **Figure 4A**). As for RR, there existed a remarkable connection between GS and BCR (p = 0.0448, **Figure 4B**). However, we did not find a significant association between publication year, follow-up duration, age, BMI value, the percentage of BMI <30 kg/m^2^, the percentage of AA, serum PSA level, GS or stage, and BCR in HR (**Figures S2A** **–I**, **Table S2**), and publication year, follow-up duration, age, BMI value, the percentage of BMI <30 kg/m^2^, the percentage of AA, serum PSA level or stage, and BCR in RR (**Figures S3A** **–H**, **Table S3**).

**Figure 5:**
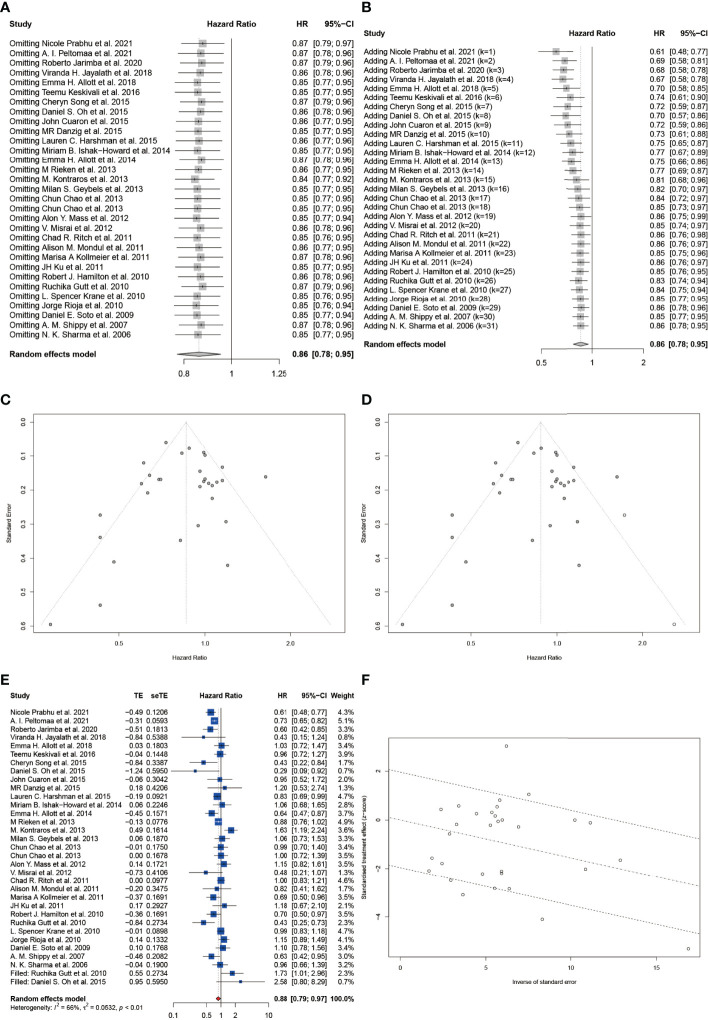
Sensitivity analysis and the detection of publication bias for included studies on HR of BCR. **(A)** Sensitivity analysis by stepwise omitting the included studies. **(B)** Cumulative meta-analysis by stepwise adding the included studies. **(C)** The funnel plot. **(D)** The trim and fill funnel plot. **(E)** The filled forest plot. **(F)** The Galbraith plot. Effect size as z-scores plotted as a function of the inverse standard error for each study reported in the present study. The middle line is the line of best fit, while the upper and lower dashed lines represent the upper and lower 95% confidence limits.

Then we conducted sensitivity analysis and cumulative meta-analysis by sequentially omitting or adding each study in turn to evaluate its effect on the pHR or pRR. For HR, we could observe that the overall estimates remained stable after omitting (**Figure 5A**) and adding (**Figure 5B**) each study. Moreover, we did not detect a statistically significant publication bias based on Begg’s test (z = -1.22, p = 0.2210) and Egger’s test (t = -0.27, p = 0.7897). Besides, the contour-enhanced funnel plot also showed good symmetry of the plot (**Figure 5C**). The trim-and-fill method suggested little evidence of publication bias (**Figure 5D**) and estimated that two studies were missing resulting from publication bias (**Figure 5E**). After filling the two missing studies, the filled forest plot also showed a significant reduction in BCR among statin users with a pHR of 0.88 (95% CI: 0.79 to 0.97, I^2^ = 66%, random effects model), which was in accordance with the original model. As shown in **Figure 5F**, the Galbraith plot also exhibited a low publication bias with most studies located between the dashed lines. As for RR, we could observe a significant change in the pooled effect when omitting or adding three studies including Oh et al., Allott et al., and Zaorsky et al. (43, 48, 56) (**Figure S4A** **, B**). The contour-enhanced funnel plot also did not show good symmetry of the plot visually with most studies lying outside of the dashed lines (**Figure S4C**). However, we did not identify a statistically significant publication bias based on Begg’s test (z = -1.14, p = 0.2550) and Egger’s test (t = -1.17, p = 0.2639). In contrast, the trim-and-fill method supported the result of the contour-enhanced funnel plot (**Figure S4D**) and estimated that two studies were missing resulting from publication bias (**Figure S4E**). After filling the two missing studies, the pooled effect lost statistical significance with a pHR of 0.83 (95% CI: 0.62 to 1.11, I^2^ = 90%, random effects model), which indicated that the publication bias might have influenced the original outcome. The Galbraith plot also exhibited a relatively high publication bias with almost half of the studies outside between the dashed lines (**Figure S4F**). Nevertheless, the L’Abbé plot showed that included studies generally agreed on the positive effect of statins in reducing the RR of BCR (**Figure S4G**).

**Figure 6:**
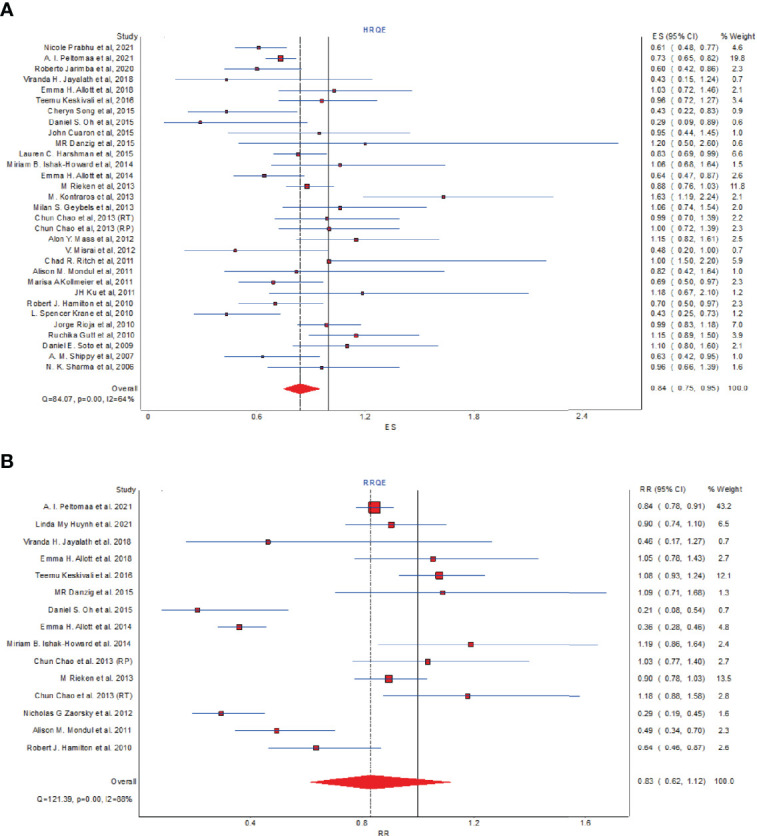
The effect of statins on BCR risk of prostate cancer among men following definitive therapy using the quality effects model. **(A)** Forest plot for the HR of BCR. **(B)** Forest plot for the RR of BCR.

Considering the quality of the included studies, we also performed meta-analyses using a QE model using the MetaXL software. As shown in **Figure 6A**, for HR, there still existed a significant reduction in BCR among statin users with study quality taken into consideration (pHR: 0.84, 95% CI: 0.75 to 0.95, I^2^ = 64%, quality effects model). However, as for RR, the result lost statistical significance when using the QE model (pHR: 0.83, 95% CI: 0.62 to 1.12, I^2^ = 88%, quality effects model, **Figure 6B**), which revealed that there existed a remarkable heterogeneity in the quality of included studies on the RR of BCR.

## Discussion

The aim of our study was to reevaluate the association between statin use and the risk of BCR among patients with PCa after definite treatment. This review comprised 33 cohort studies, including 31 studies reporting the HR of BCR and 15 studies reporting the RR of BCR. We found that statin use was tightly connected with the reduction of BCR among patients with PCa, especially for those accepting RT as their primary treatment, which was consistent with previously published meta-analyses (33, 76). Although patients in the subgroup accepting ADT also showed a significant reduction in HR of BCR, the number of studies in this subgroup was too limited and part of the patients have also accepted other treatments except ADT in these studies, which could bring bias into the final results. Although five studies have reported a remarkable effect of statins on the reduction of BCR after RP, the pooled effect showed no statistical significance due to the heterogeneity of included studies. An RCT study published by Jeong et al. in 2021 showed that 20 mg/day of atorvastatin use for 24 months had no significant effect on the risk of BCR in patients with high-grade prostate cancer after RP (HR, 1.00; 95% CI, 0.71–1.41), which was in accordance with our conclusion. The reason why statins could reduce the risk of BCR could be that statin might improve the radiosensitivity of prostate cancer by causing cell-cycle arrest in the late G1 phase (77). Statins could induce late G1 arrest and apoptosis by inhibition of cdk2, E2F1, p21, and/or p27 (78). A recent study showed that statins could also enhance the effects of RT by triggering the interaction between Bcl-2 and MSH2 (79) and compromising DNA double-strand breaks repair (80). The development and progression of PCa were dependent on androgens, and cholesterol is a precursor for androgen synthesis. Therefore, cholesterol lowering by statins could suppress androgen synthesis and enhance the efficacy of ADT treatment. It was also reported that statins could compete with androgens for influx by the SLCO2B1 transporter, thus decreasing tumor’s androgen supply (81). *In vitro* studies have also discovered that statins could increase the therapeutic effect of abiraterone acetate and enzalutamide (82). Further studies are needed, and more clinical trials should be carried out to verify the hypotheses.

Previous epidemiological observations and preclinical models suggested that hypercholesterolemia might play a crucial role in the incidence and progression of PCa, especially in increasing the risk of high-grade, aggressive disease and castration resistance (83, 84). It was also reported that elevated cholesterol was associated with increased risk of recurrence among men with dyslipidemia after RP (75). In our study, using meta-regression, we found that serum cholesterol level was a significant confounder which could neutralize the protective effect of statins on BCR, which indicated that statins might reduce the risk of BCR by mediating hypercholesterolemia. A recent study has found that sterol-O-acyl transferases (SOAT) 1, an enzyme involved in cholesteryl ester synthesis, was remarkably connected to earlier BCR in high-risk prostate cancer (85). However, Lefebvre et al. observed that there existed no significant association between metabolic syndrome including hypercholesterolemia and the risk of BCR in Afro-Caribbean men with PCa after RP (86). Allott et al. also found that high cholesterol was not associated with progression of PCa after RP or RT (40). Therefore, it was still controversial and more studies were needed.

We also observed that serum PSA level was significantly lower in statin users compared with non-users in many included studies (46, 57, 59, 62, 65). It was reported that PSA level could be influenced by smoking status, Gleason score, and 5α-reductase inhibitor for benign prostate hyperplasia treatment, but not associated with other clinical factors including hypercholesterolemia in a retrospective study (87, 88). An RCT study published by Murtola et al. in 2018 showed that 80 mg/day of atorvastatin could not significantly reduce the tumor proliferation index (Ki-67) and PSA level, but in subgroup analyses, atorvastatin use over 28 days exhibited a significant reduction in Ki-67 and PSA (89). Therefore, it was possible that statin use could only cover the truth of BCR and disease progression through decreasing the PSA level instead of preventing BCR. However, if this hypothesis was true, the detection of BCR would be delayed and the prognosis would be worse. However, the previously published meta-analysis found that statins have a significant effect on the reduction of tumor metastasis, all-cause mortality, and PCSM after treatment (33), which indicated a better prognosis and was contradictory to this hypothesis.

In this review, we have also used various methods to detect, evaluate, and diminish the probable heterogeneity and publication bias of included studies. For studies about the HR of BCR, the sensitivity analysis and cumulative meta-analysis all showed a stable pooled result. The funnel plot, Begg’s test, and Egger’s test all exhibited little publication bias from qualitative and quantitative perspectives, respectively. After the trim-and-fill method, the pooled result also had a statistical significance. However, as for studies about the RR of BCR, there did exist a relatively high heterogeneity and publication bias. This could result from the limited number of included studies, and RR did not take time into consideration, which could lead into bias in the methodology. We also used the QE model, which took the quality of studies into account, to reevaluate the pooled results of RR and HR. Not surprisingly, the pHR remained stable with a statistical significance, which further proved that our results were religious and authentic.

Nevertheless, there still existed many limitations in our review. First, the definitions of statin use were various in the included studies. Information about the types of statins, the duration of statin use, the dose of statins, and the initiation of statin use (before or after primary treatment) was not complete and detailed in the included studies. Therefore, we could not take this into consideration, which will definitely contribute to the heterogeneity of studies. Second, there existed great heterogeneity in the characteristics of the studying cohort. Many patients in the statin group had preexisting comorbidities such as cardiovascular diseases and metabolic syndrome, which could influence the progression of PCa. Third, the characteristics of PCa could also be a potential confounder of the results. Although tumor stage did not show a statistical significance in the meta-regression, the GS, metastasis status, PCa volume, and surgical margin status could all be connected with BCR and contribute to the heterogeneity of studies. Fourth, although we have performed subgroup analyses according to the primary treatment, many patients did not accept only one kind of treatment and part of patients also accepted ADT after RT or RP, which could interfere with the result of subgroup analyses. Fifth, although many studies have provided the results adjusted for important covariates, some unadjusted results might influence the final pooled effect. Finally, although the pHR showed that statins lowered the BCR of PCa, the upper confidence interval was close to 1.00. Thus, the result needs to be deliberately explained.

In conclusion, despite some limitations, our study suggests that statin, a widely used and relatively cheap drug, has a unique role in the reduction of BCR in patients with PCa after definite treatment, especially RT. In the future, more clinical trials and *in vitro* and animal experiments were needed to further verify the effects of statins in PCa and the mechanisms behind this phenomenon.

## Data Availability Statement

The original contributions presented in the study are included in the article/**Supplementary Material**. Further inquiries can be directed to the corresponding authors.

## Author Contributions

J-XS, X-YZ, Z-BZ, Q-DX, and S-GW contributed to developing the main research question, carrying out the literature search, collecting the included studies’ information, and describing the results. J-XS performed the meta-analysis and wrote the first draft of the manuscript. C-QL and J-ZX contributed to developing the main research question and revised the manuscript. YA, M-YX, and JH revised the manuscript. All authors contributed to the article and approved the submitted version. J-XS, Z-BZ, Q-DX, and S-GW contributed equally to this work.

## Funding

This work was supported by the Natural Science Foundation of China (81772729) and Undergraduate Training Program for Innovation and Entrepreneurship (DELC2022010).

## Conflict of Interest

The authors declare that the research was conducted in the absence of any commercial or financial relationships that could be construed as a potential conflict of interest.

## Publisher’s Note

All claims expressed in this article are solely those of the authors and do not necessarily represent those of their affiliated organizations, or those of the publisher, the editors and the reviewers. Any product that may be evaluated in this article, or claim that may be made by its manufacturer, is not guaranteed or endorsed by the publisher.

## Supplementary Material

Supplementary Figure 1 The forest plot for subgroup analyses. **(A)** The forest plot for the RR of BCR with subgroup analyses by primary treatment. **(B)** The forest plot for the RR of BCR with subgroup analyses by country. **(C)** The forest plot for the HR of BCR with subgroup analyses by country.

Supplementary Figure 2 The meta-regression for HR of BCR and covariates. **(A)** Tumor stage. **(B)** GS. **(C)** PSA. **(D)** The percentage of AA. **(E)** BMI<30. **(F)** BMI value. **(G)** Age. **(H)** Follow-up duration. **(I)** Publication year. Each dot represents an individual study. Symbol size represents sample size.

Supplementary Figure 3 The meta-regression for RR of BCR and covariates. **(A)** BMI<30. **(B)** BMI value. **(C)** PSA. **(D)** Tumor stage. **(E)** Publication year. **(F)** Age. **(G)** Follow-up duration. **(H)** The percentage of AA. Each dot represents an individual study. Symbol size represents sample size.

Supplementary Figure 4 Sensitivity analysis and the detection of publication bias for included studies on RR of BCR. **(A)** Sensitivity analysis by stepwise omitting the included studies. **(B)** Cumulative meta-analysis by stepwise adding the included studies. **(C)** The funnel plot. **(D)** The trim and fill funnel plot. **(E)** The filled forest plot. **(F).** The Galbraith plot. Effect size as z-scores plotted as a function of the inverse standard error for each study reported in the present study. The middle line is the line of best fit, while upper and lower dashed lines represent the upper and lower 95% confidence limits. **(G)** The L’Abbé plot for incidence of BCR. Each dot represents an individual study. Symbol size represents sample size.

Supplementary Table 3 Univariable meta-regression for the RR of biochemical recurrence.
