# Supplementary figures and images for: Statin Use and the Risk of Prostate Cancer Biochemical Recurrence Following Definitive Therapy: A Systematic Review and Meta-Analysis of Cohort Studies
Source: Front Oncol. 2022 May 9;12:887854. doi: 10.3389/fonc.2022.887854 (PMC9124863; doi:10.3389/fonc.2022.887854)

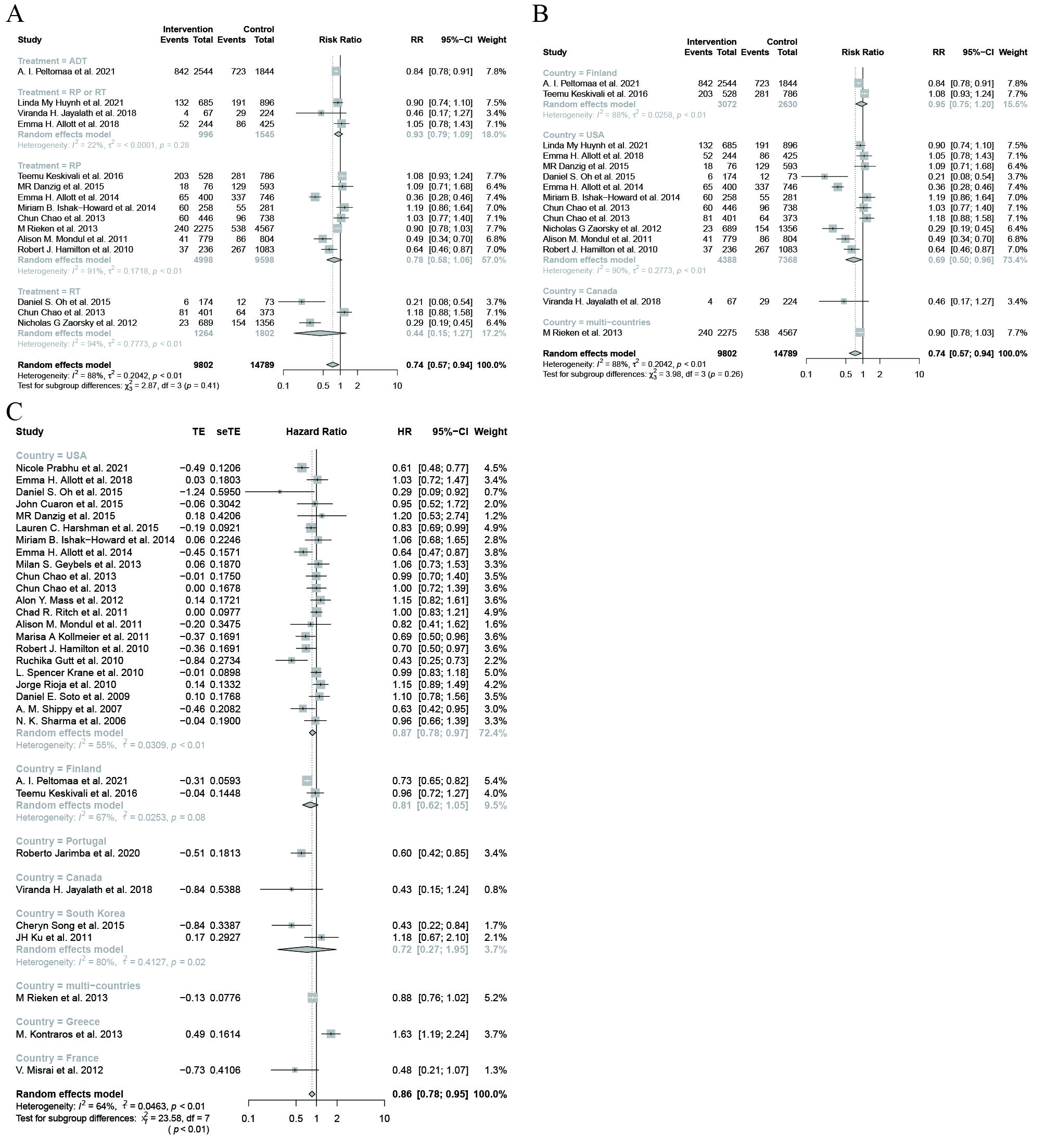

Supplement: Supplementary Figure 1 — The forest plot for subgroup analyses. (A) The forest plot for the RR of BCR with subgroup analyses by primary treatment. (B) The forest plot for the RR of BCR with subgroup analyses by country. (C) The forest plot for the HR of BCR with subgroup analyses by country. [file Image_1.jpeg]

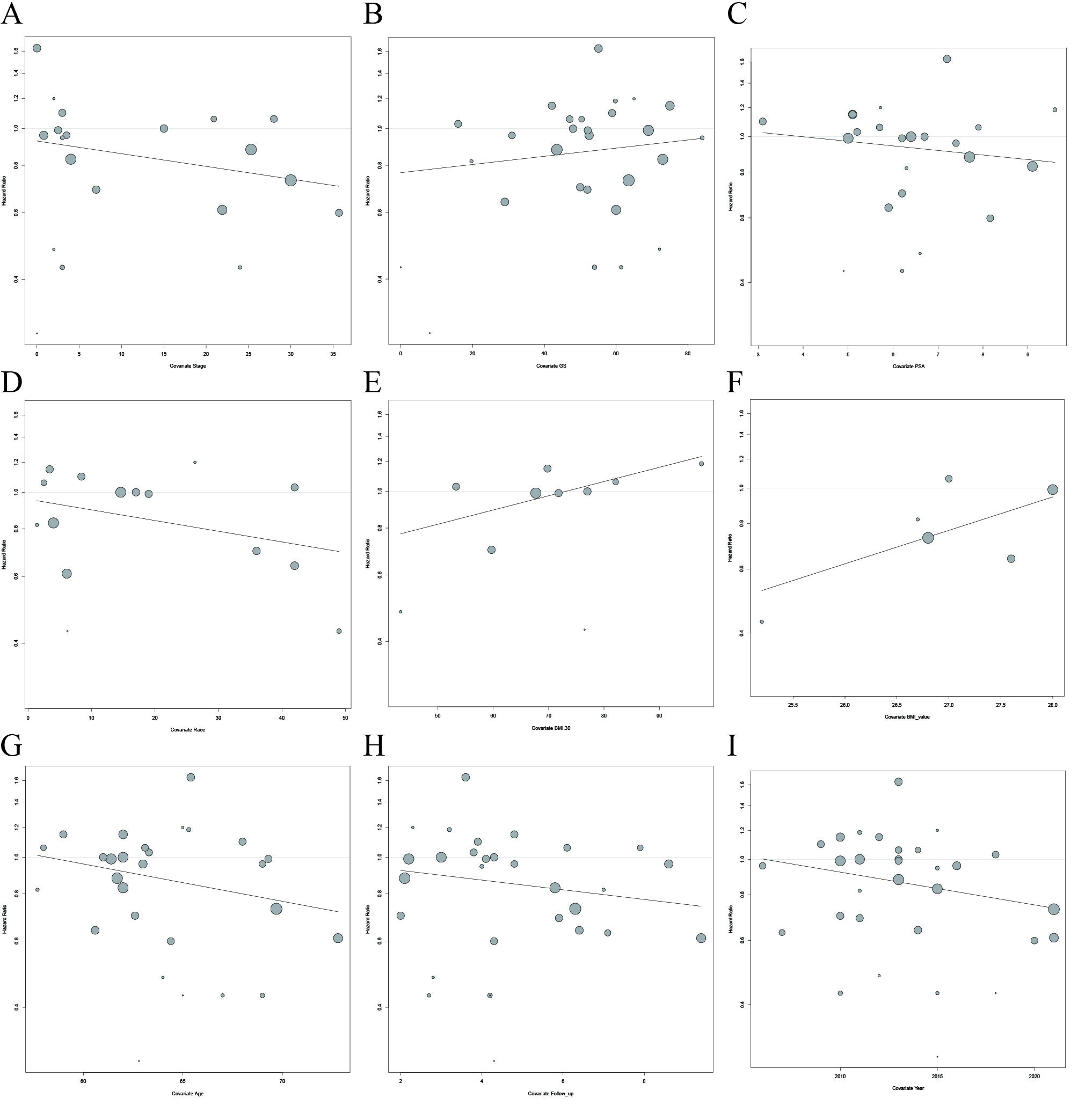

Supplement: Supplementary Figure 2 — The meta-regression for HR of BCR and covariates. (A) Tumor stage. (B) GS. (C) PSA. (D) The percentage of AA. (E) BMI<30. (F) BMI value. (G) Age. (H) Follow-up duration. (I) Publication year. Each dot represents an individual study. Symbol size represents sample size. [file Image_2.jpeg]

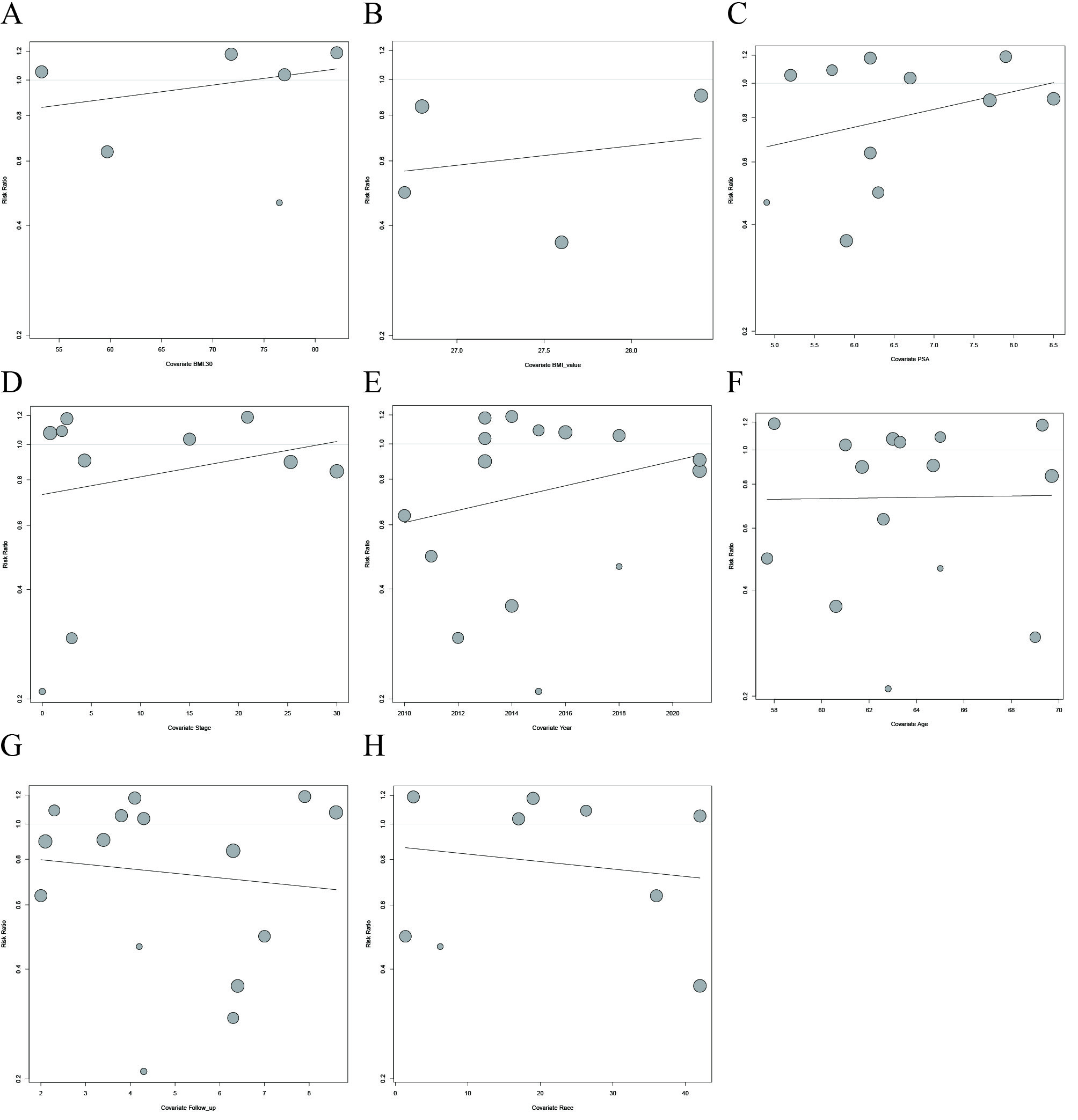

Supplement: Supplementary Figure 3 — The meta-regression for RR of BCR and covariates. (A) BMI<30. (B) BMI value. (C) PSA. (D) Tumor stage. (E) Publication year. (F) Age. (G) Follow-up duration. (H) The percentage of AA. Each dot represents an individual study. Symbol size represents sample size. [file Image_3.jpeg]

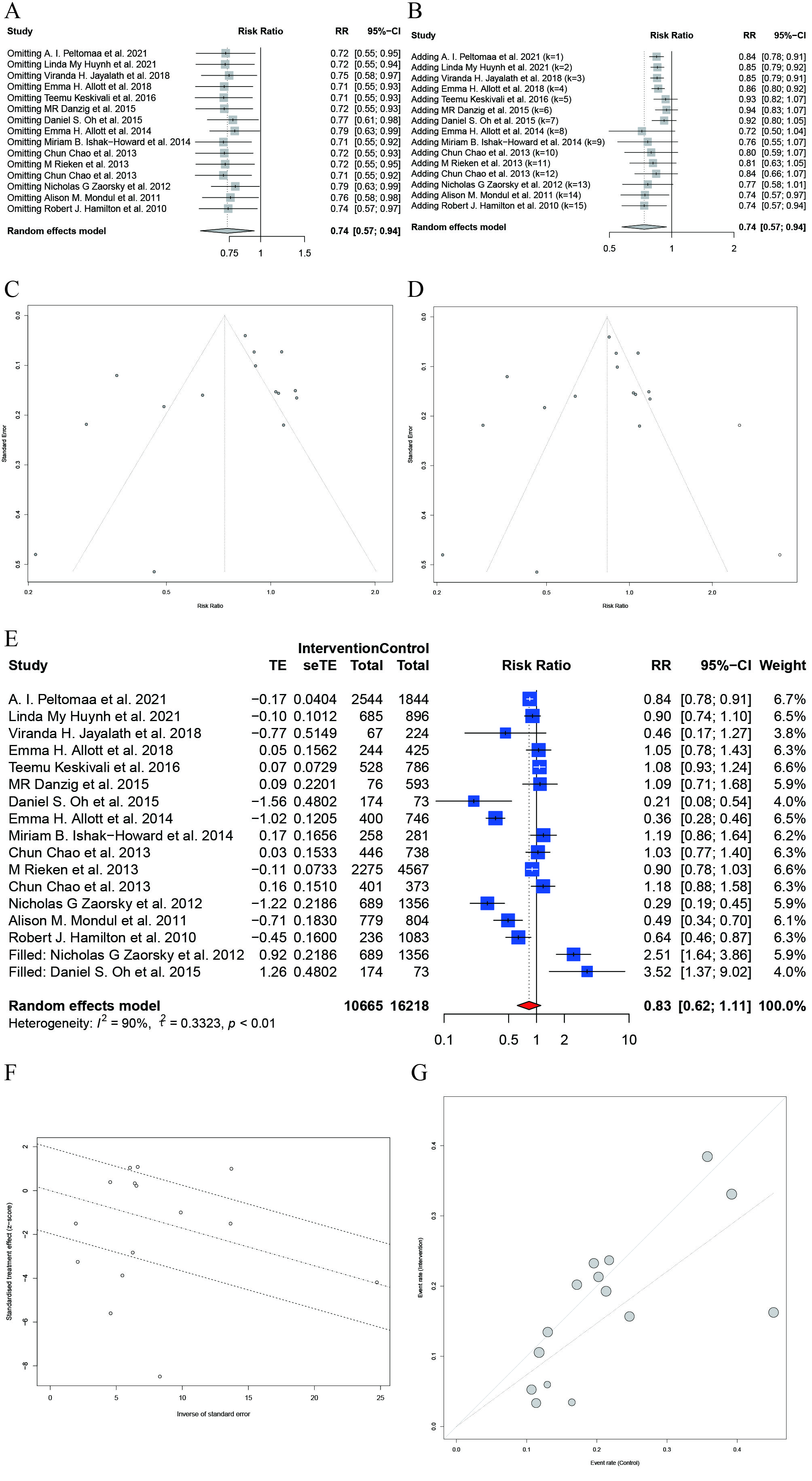

Supplement: Supplementary Figure 4 — Sensitivity analysis and the detection of publication bias for included studies on RR of BCR. (A) Sensitivity analysis by stepwise omitting the included studies. (B) Cumulative meta-analysis by stepwise adding the included studies. (C) The funnel plot. (D) The trim and fill funnel plot. (E) The filled forest plot. (F). The Galbraith plot. Effect size as z-scores plotted as a function of the inverse standard error for each study reported in the present study. The middle line is the line of best fit, while upper and lower dashed lines represent the upper and lower 95% confidence limits. (G) The L’Abbé plot for incidence of BCR. Each dot represents an individual study. Symbol size represents sample size. [file Image_4.jpeg]
