# Supplementary material for: Statin Use and the Risk of Prostate Cancer Biochemical Recurrence Following Definitive Therapy: A Systematic Review and Meta-Analysis of Cohort Studies
Source: Front Oncol. 2022 May 9;12:887854. doi: 10.3389/fonc.2022.887854 (PMC9124863; doi:10.3389/fonc.2022.887854)
Supplement: Supplementary file 5 [file Table_1.docx]

**Table S1A: Details of Search Strategy to Retrieve the Studies using PubMed (Medline)**

**Date of Search: 12/31/2021**

| **#** | **Search Terms** | **Hits** |
| --- | --- | --- |
| #1 | (((((((((((Statin[Title/Abstract]) OR (Atorvastatin[Title/Abstract])) OR (Cerivastatin[Title/Abstract])) OR (Compactin[Title/Abstract])) OR (Fluvastatin[Title/Abstract])) OR (HMG-CoA[Title/Abstract])) OR (Lovastatin[Title/Abstract])) OR (Mevastatin[Title/Abstract])) OR (Pravastatin[Title/Abstract])) OR (Rosuvastatin[Title/Abstract])) OR (Rosvastatin[Title/Abstract])) OR (Simvastatin[Title/Abstract]) | 48823 |
| #2 | Search: ((((((((((((((((Prostatic Neoplasms[Title/Abstract]) OR (Prostate Neoplasms[Title/Abstract])) OR (Neoplasms, Prostate[Title/Abstract])) OR (Neoplasm, Prostate[Title/Abstract])) OR (Prostate Neoplasm[Title/Abstract])) OR (Neoplasms, Prostatic[Title/Abstract])) OR (Neoplasm, Prostatic[Title/Abstract])) OR (Prostatic Neoplasm[Title/Abstract])) OR (Prostate Cancer[Title/Abstract])) OR (Cancer, Prostate[Title/Abstract])) OR (Cancers, Prostate[Title/Abstract])) OR (Prostate Cancers[Title/Abstract])) OR (Cancer of the Prostate[Title/Abstract])) OR (Prostatic Cancer[Title/Abstract])) OR (Cancer, Prostatic[Title/Abstract])) OR (Prostatic Cancers[Title/Abstract])) OR (Cancer of Prostate[Title/Abstract]) | 136233 |
| **#3** | **#1 AND #2** | **403** |

**Table S1B: Details of Search Strategy to Retrieve the Studies using Embase**

**Date of Search: 12/31/2021**

| **#** | **Search Terms** | **Hits** |
| --- | --- | --- |
| #1 | (Statin or Atorvastatin or Cerivastatin or Compactin or Fluvastatin or HMG-CoA or Lovastatin or Mevastatin or Pravastatin or Rosuvastatin or Rosvastatin or Simvastatin).ab,kw,ti. | 77591 |
| #2 | (prostate cancer or prostatic cancer or prostate gland cancer or cancer, prostate).ab,kw,ti. | 200184 |
| **#3** | **#1 AND #2** | **718** |

**Table S1C: Details of Search Strategy to Retrieve the Studies using Cochrane**

**Date of Search: 12/31/2021**

| **#** | **Search Terms** | **Hits** |
| --- | --- | --- |
| #1 | (Statin):ti,ab,kw or(Atorvastatin):ti,ab,kw or(Cerivastatin):ti,ab,kw or(Compactin):ti,ab,kw or(Fluvastatin):ti,ab,kw or(HMG-CoA):ti,ab,kw or(Lovastatin):ti,ab,kw or(Mevastatin):ti,ab,kw or(Pravastatin):ti,ab,kw or(Rosuvastatin):ti,ab,kw or(Rosvastatin):ti,ab,kw or(Simvastatin):ti,ab,kw | 15809 |
| #2 | (Prostatic Neoplasms):ti,ab,kw or(Prostate Neoplasms):ti,ab,kw or(Neoplasms, Prostate):ti,ab,kw or(Neoplasm, Prostate):ti,ab,kw or(Prostate Neoplasm):ti,ab,kw or(Neoplasms, Prostatic):ti,ab,kw or(Neoplasm, Prostatic):ti,ab,kw or(Prostatic Neoplasm):ti,ab,kw or(Prostate Cancer):ti,ab,kw or(Cancer, Prostate):ti,ab,kw or(Prostate Cancers):ti,ab,kw or(Cancers, Prostate):ti,ab,kw or(Cancer of the Prostate):ti,ab,kw or(Prostatic Cancer):ti,ab,kw or(Cancer, Prostatic):ti,ab,kw or(Cancer of Prostate):ti,ab,kw or(Prostatic Cancers):ti,ab,kw | 15186 |
| **#3** | **#1 AND #2** | **81** |
