# Supplementary material for: Statin Use and the Risk of Prostate Cancer Biochemical Recurrence Following Definitive Therapy: A Systematic Review and Meta-Analysis of Cohort Studies
Source: Front Oncol. 2022 May 9;12:887854. doi: 10.3389/fonc.2022.887854 (PMC9124863; doi:10.3389/fonc.2022.887854)
Supplement: Supplementary file 6 [file Table_2.docx]

**Table S2 Univariable meta-regression for the HR of biochemical recurrence.**

| **Covariates** | **Coefficient (beta)** | **95% CI** | **P-value** |
| --- | --- | --- | --- |
| Publication year | -0.0207 | (-0.0454, 0.0041) | 0.1015 |
| Follow-up duration | -0.0296 | (-0.0783, 0.0191) | 0.6488 |
| Age | -0.0228 | (-0.0499, 0.0043) | 0.0992 |
| BMI value | 0.2111 | (-0.0429, 0.4651) | 0.1034 |
| BMI<30 | 0.0087 | (-0.0026, 0.0199) | 0.1298 |
| Cholesterol** | 0.0308 | (0.0083, 0.0533) | 0.0074 |
| AA% | -0.0065 | (-0.0153, 0.0023) | 0.149 |
| PSA | -0.0287 | (-0.0993, 0.0420) | 0.4265 |
| GS | 0.0025 | (-0.0042, 0.0092) | 0.4671 |
| Tumor stage≥T3 | -0.0077 | (-0.0186, 0.0032) | 0.1642 |

**Abbreviations: CI, confidence interval; BMI, body mass index; AA, African American; PSA, prostate specific antigen; GS, Gleason score.**

****: P < 0.01**
