# Supplementary material for: Statin Use and the Risk of Prostate Cancer Biochemical Recurrence Following Definitive Therapy: A Systematic Review and Meta-Analysis of Cohort Studies
Source: Front Oncol. 2022 May 9;12:887854. doi: 10.3389/fonc.2022.887854 (PMC9124863; doi:10.3389/fonc.2022.887854)
Supplement: Supplementary Table 3 — Univariable meta-regression for the RR of biochemical recurrence. [file Table_3.docx]

**Table S3 Univariable meta-regression for the RR of biochemical recurrence.**

| **Covariates** | **Coefficient (beta)** | **95% CI** | **P-value** |
| --- | --- | --- | --- |
| Publication year | 0.0387 | (-0.0361, 0.1136) | 0.3102 |
| Follow-up duration | -0.0286 | (-0.1519, 0.0946) | 0.6488 |
| Age | 0.0022 | (-0.0689, 0.0733) | 0.9524 |
| BMI value | 0.1221 | (-0.6393, 0.8835) | 0.7533 |
| BMI<30 | 0.0084 | (-0.0133, 0.0302) | 0.447 |
| AA% | -0.0047 | (-0.0259, 0.0165) | 0.6623 |
| PSA | 0.1157 | (-0.1209, 0.3523) | 0.3377 |
| GS* | 0.0116 | (-0.0171, 0.0395) | 0.0448 |
| Tumor stage≥T3 | 0.0112 | (0.0003, 0.0231) | 0.4392 |

**Abbreviations: CI, confidence interval; BMI, body mass index; AA, African American; PSA, prostate specific antigen; GS, Gleason score.**

***: P < 0.05**
